# Recent Advances in Moisture‐Electric Nanogenerators: From Moisture‐Enabled Electrification to Practical Applications

**DOI:** 10.1002/smll.202509502

**Published:** 2026-02-22

**Authors:** Xiangming Dai, Nathaniel Corrigan, Cyrille Boyer, Dewei Chu, Jin Zhang

**Affiliations:** ^1^ School of Mechanical and Manufacturing Engineering Ainsworth Building University of New South Wales Sydney New South Wales Australia; ^2^ Centre For Advanced Manufacturing Technology (CfAMT) School of Engineering Design and Built Environment Western Sydney University Sydney New South Wales Australia; ^3^ School of Chemical Engineering Building E8 UNSW Sydney New South Wales Australia; ^4^ School of Materials Science and Engineering Hilmer Building University of New South Wales Sydney New South Wales Australia

**Keywords:** electrical output, moisture‐electric generation, nanomaterials, sustainable energy

## Abstract

The global energy shortage continues to raise energy prices and cause an imbalance between supply and demand of oil, gas and electricity. This ongoing worldwide energy crisis highlights the urgent need for exploiting more renewable and clean energy from natural resources while simultaneously minimizing the carbon footprint. Moisture‐electric nanogenerators (MEGs) have emerged as a novel method for energy harvesting, utilizing the ubiquity, sustainability, and portability of atmospheric moisture, and overcoming regional restrictions for thermal, solar, or mechanical energy inputs. By exchanging intermolecular bonding energy when ionizing moisture into electrical output through deliberately fabricated hygroscopic materials, MEGs can have diverse applications, including self‐powered sensors and low‐power sources used for humidity sensing, respiration monitoring, etc. This review covers the construction, materials, and mechanisms of the MEGs, the recent progress in cutting‐edge innovations in moisture‐responsive materials for boosting electrical outputs, followed by discussions of practical MEG applications. The outlook for further development of MEGs is also provided, along with the predicted increase in use cases of this promising clean energy‐harvesting approach.

## Graphical abstract

This review covers the construction, materials, and mechanisms of the moisture‐electric nanogenerators (MEGs), the recent progress in cutting‐edge innovations in moisture‐responsive materials for boosting electrical outputs, followed by discussions of practical MEG applications. The outlook for further development of MEGs is also provided, along with the predicted increase in use cases of this promising clean energy‐harvesting approach.

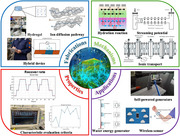

## 1 Introduction

The continuous developments of self‐powered sensors, wearable electronics, and advanced energy supply systems have highlighted the critical demand for sustainable and autonomous power sources. Conventional batteries suffer from constraints including limited lifespans, environmental toxicity, high rigidity, and heavy reliance on periodic replacement or recharging. These limitations have propelled studies into ambient energy harvesting technologies such as solar cells, thermoelectric generators, and piezoelectric transducers, which can convert environmental inputs into electrical outputs. Among emerging alternative energy harvesting technologies, moisture‐electric nanogenerators (MEGs) have attracted increasing attention in the past five years. These nanogenerators can harvest ubiquitous water molecules from humid atmospheric air or by direct moisture contact to generate stable electrical outputs through the conversion of chemical potential energy of atmospheric water vapor [1, 2].

MEGs depend on the interaction between water molecules and functional materials, causing ion dissociation and diffusion that produces a flow of current. The basis involves creating a concentration gradient of ions at a water‐solid interface and guiding them to transport and generate a potential difference. Unlike solar and wind power, which require specific environmental conditions, MEGs operate continuously under a wide range of moisture conditions, offering reliable applications in both indoor and outdoor settings. Their intrinsic advantages include light weight from the membrane structure [3], technological scalability, environmental compatibility, and low operational costs. These features strongly position MEGs to power self‐sustained devices within the fields of health monitoring, environmental sensing, and smart infrastructure [4, 5].

As MEG electrochemical processes are regulated by complex interactions between water molecules and nanostructured materials, construction and materials are critical in enabling efficient ion dissociation and directional ion diffusion, and thus generating electrochemical potential gradients. Advancements in these two domains are usually synergistically integrated. In this regard, material choices, for instance, hydrophilic polymers, graphene oxide [6], carbon nanocomposites [7], metal oxide and metal hydroxide nanomaterials [8], hydrogels [9], aerogels [10, 11] and biomaterials [12] are frequently employed to create nanochannels and heterogeneous interfaces that facilitate proton transport and enhance charge separation efficiency. Fabrication methods include constructing asymmetric polyelectrolyte layers [1], electrospinning for nanofibre membranes [13], and synthesizing 3D porous graphene‐based aerogels [5] and supramolecular hydrogels [9, 14]. These approaches enable profound moisture absorption, increase surface charge density, and stabilize ion migration across varied humidity conditions. Moreover, heterojunction‐based designs, such as graphene oxide (GO)/reduced GO (rGO) hybrid membranes [5, 6], metal oxide‐carbon nanotube composites [8], and layered hydrophilic‐hydrophobic interfaces have demonstrated their superiority in enhancing ion diffusion and operational stability. The synergistic effects between morphological structure and material electrochemistry determine the ability of MEGs to achieve high output performance.

Exploring how material structures interact with MEG mechanisms to enhance moisture‐electric conversion has become an important emerging research topic. Analyzing the underlying fundamental mechanisms can guide the rational design of more optimized morphologies and structures in hygroelectric composites. Since seminal studies around 2015, a great deal of research attention has been given to the mechanisms of MEG ion dissociation and diffusion from hygroscopic functional groups, electrokinetic streaming potentials through nanochannels and special electrode pairs [15], and electric double‐layer (EDL) formation at interfaces [10]. In many MEG devices, moisture‐induced gradients in ion concentration and proton mobility can establish a continuous electric field. Recent mechanistic innovations have enabled hybrid systems that combine MEGs with complementary energy transition processes, including metal–air redox reactions [1, 16], triboelectric and piezoelectric effects [17, 18], and photocatalysis [19]. The multi‐functional platforms not only improve energy density and reliability but also extend MEG applications to more dynamic and extreme environments.

The integration of these hybrid mechanisms has accelerated progress toward multifunctional power‐supply systems. By working with additional energy conversion pathways, such as vibrational, thermal, and photoactive energies, moisture‐induced hybrid MEGs can achieve higher output performance and longer lifespans. For example, systems that integrate triboelectric nanogenerators (TENGs) or piezoelectric actuators with MEGs can realize the function of generating both continuous and pulsed electrical signals [20]. In addition, biologically derived materials, such as protein nanofibrils [21] and alginate hydrogels [13], are beneficial in terms of their eco‐friendly fabrication methods and biodegradability while maintaining the expected electrical characteristics. Currently, the most promising industry‐compatible techniques that are being developed include developed hydrogel‐based MEGs and incorporating hydrogel MEGs to form hybrid energy harvesting or complementary modes. These techniques can utilize self‐adaptable capacity and higher ion transport of hydrogel to realize structural flexibility as well as to minimize the energy loss during body motions. The hybrid system can even increase the electrical outputs within the complementary modes.

Despite continuous improvements in developing more promising industry‐compatible MEGs, challenges remain in various application scenarios. Scaling fabrication processes, ensuring device stability in fluctuating humidity environments, and achieving consistent power density across diverse application contexts are unresolved technical problems in the areas of self‐powered MEGs. On the other hand, moisture‐electric material research has increasingly focused on sustainable sources, facile synthesis methods, and nanostructure engineering to overcome the limitations of overconsumed non‐renewable energy sources. Environmentally nontoxic materials from renewable sources are being incorporated to refine both output performances and green fabrication processes [21, 22]. Unique scientific questions are raised when considering the existing challenges, e.g., What fundamental relationships of material, structure, and mechanism govern efficient MEGs? How do different MEG designs solve the limitations in ion transport dynamics and electrical output? What are the most effective strategies for enhancing MEG performance? How can MEGs transition from laboratory demonstrations to scalable industry‐compatible technologies? The broad applications of MEGs are distinct across a range of domains, from autonomous biomedical sensors, humidity‐responsive wearable electronics, to building‐integrated energy harvesting systems and moisture‐powered environmental diagnostics [23, 24, 25, 26]. Furthermore, their integration into internet of things (IoTs) platforms holds significant potential for battery‐free sensor networks and arrayed monitoring systems [25, 26].

**FIGURE 1:**
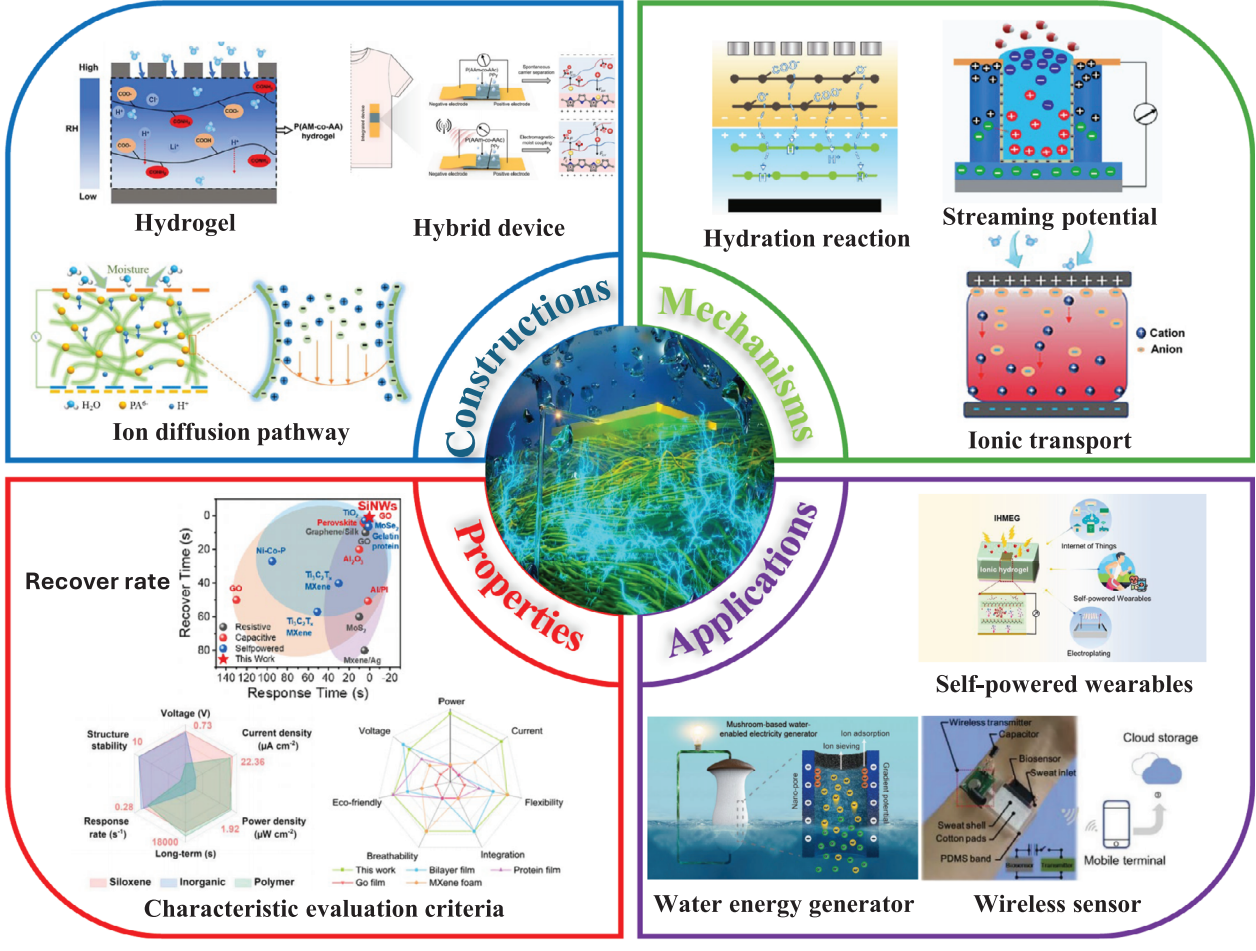
Overview of MEGs. Constructions and materials: Hydrogel concept. Reproduced with permission [27]. Copyright 2025, Cell Press. Ion diffusion pathway. Reproduced with permission [13]. Copyright 2024, Elsevier. Hybrid device. Reproduced with permission [28]. Copyright 2025, Springer Nature, under the terms of Creative Commons CC BY 4.0 license. Mechanisms: Hydration reaction. Reproduced with permission [5]. Copyright 2023, Elsevier. Streaming potential. Reproduced with permission [15]. Copyright 2023, Elsevier. Ionic transport. Reproduced with permission [27]. Copyright 2025, Elsevier. Properties: Recover rate. Reproduced with permission [15]. Copyright 2023, Elsevier. Characteristic evaluation criteria. Reproduced with permission [10, 29]. Copyright 2024, Springer Nature, under the terms of Creative Commons CC BY 4.0 license; Copyright 2024, Elsevier. Applications: Self‐powered wearables. Reproduced with permission [30]. Copyright 2022, Elsevier. Water energy generator. Reproduced with permission [31]. Copyright 2024, ACS Publications. Wireless sensor. Reproduced with permission [32]. Copyright 2022, Elsevier.

Compared with previous reviews that summarized hydrovoltaic or moisture‐induced electricity generation in broad terms, this work differs in both scope and analytical depth. The work uniquely integrates MEG mechanisms with a systematic classification of fabrications, structure‐property relationships, and energy conversion pathways. It emphasizes new hygroscopic materials, nano architecture, optimized electrodes, supplementary energy conversion methods that can raise electrical outputs, and materialize scalable, durable, and multifunctional MEG systems for next‐generation green energy harvesting applications that are not captured in previous summaries. Moreover, it is the first time that a consolidated discussion of performance enhancement strategies within a unified MEG framework is provided. By mapping these developments to emerging application fields and future industrial opportunities, this work offers a differentiated and targeted perspective that can extend beyond existing MEG applications (Figure 1).

## 2 Constructions and Materials

**FIGURE 2:**
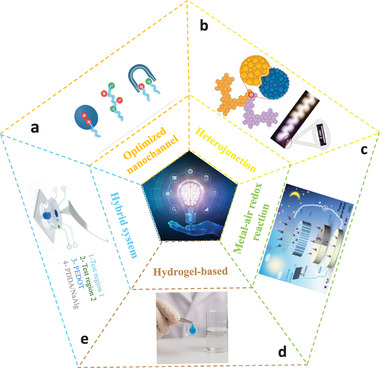
Constructions and materials for high‐performance MEGs. (a) Optimization in ion transmission pathways by creating nanochannels. (b) Heterojunctions. Reproduced with permission [33]. Copyright 2023, under the terms of the Full Beilstein‐Institut Open Access License Agreement 1.2. (c) Metal–air redox reaction. Reproduced with permission [25]. Copyright 2024, Wiley, under the terms of Creative Commons CC BY 4.0 license. (d) Hygroscopic hydrogels and aerogels. (e) Hybrid systems which can induce synergistic effects. Reproduced with permission [10]. Copyright 2024, Springer Nature, under the terms of Creative Commons CC BY 4.0 license.

The diverse moisture‐enabled electricity generation modes in MEGs are a result of different material architectures and energy conversion mechanisms. Figure 2 provides a simple overview of construction and materials for high‐performance MEGs. Nanochannels can be programmed and increased to optimize ion transport pathways, enabling enhanced charge separation and directional proton migration under the moisture gradient. Charge‐selective pores, asymmetric confinement, and other nano‐structural features can function to form pervasive nanochannels and facilitate high output performance in MEGs [15] (Figure 2a). Heterojunctions are usually formed between dissimilar materials with asymmetric zeta potential, surface chemistry, moisture absorption, and water/ionic percentage, which cause junction potential that predominantly contributes to electricity generation [6] (Figure 2b). A metal–air redox system can complement the physical moisture‐induced ion migration (Figure 2c), by boosting carrier diffusion speed to facilitate ion diffusion, which has been widely applied in health monitoring wearable devices [25]. Hygroscopic hydrogels (Figure 2d), including those based on sulfobetaine methacrylate (SBMA), polyacrylamide (PAM) or polyvinyl alcohol (PVA), critically emphasized their importance in moisture harvesters. Through storing or transporting moisture, they can activate ion dissociation and regulate diffusion, while maintaining excellent structural flexibility and autonomous self‐healing capacity. Hybrid moisture‐electric systems (Figure 2e) are mostly fabricated as a stacked device composed of multilayer materials with different functions such as triboelectric, piezoelectric, or thermoelectric properties, to synergistically enhance the output performance. These combinations enable multimodal energy conversion, such as concurrent moisture and mechanical energy harvesting with additional environmental adaptability.

### 2.1 Optimization in Ion Transmission Pathways

**FIGURE 3:**
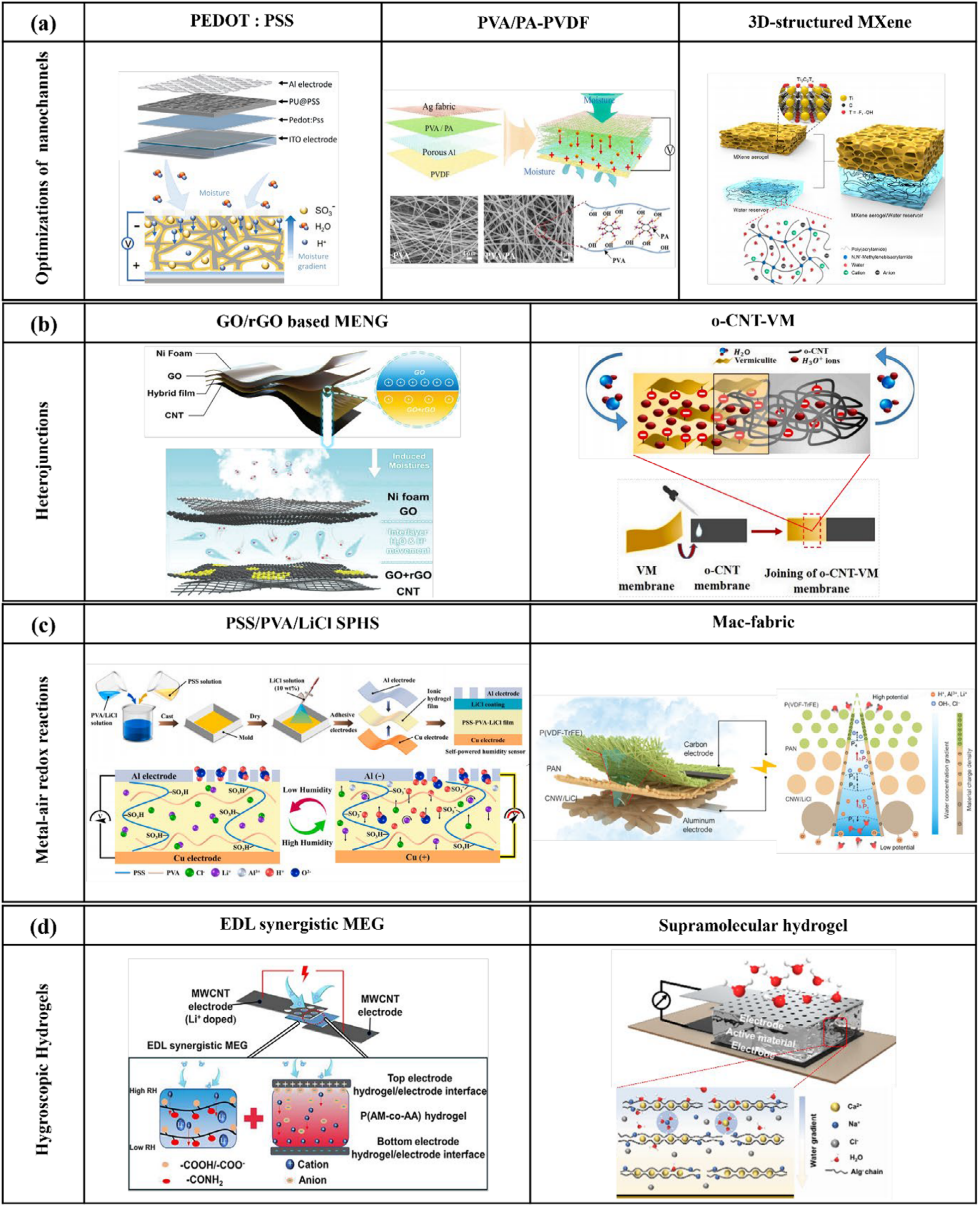
Examples of constructions and materials for high performance MEGs. (a) Synergistic nanoarchitecture based on PEDOT: PSS interception layer (left). Reproduced with permission [2]. Copyright 2023, Elsevier. Hydrophilic‐hydrophobic nanofibre membrane (PVA/PA‐PVDF) (middle). Reproduced with permission [13]. Copyright 2024, Elsevier. 3D‐structured MXene aerogel film stacked on PAM hydrogel (right). Reproduced with permission [4]. Copyright 2023, ACS Publications. (b) Heterogeneous designs for creating heterojunctions. e.g. GO/rGO MENGs (left) and MENG based on o‐CNT‐VM membrane (right). Reproduced with permission [5, 6]. Copyrights 2023 and 2024, Elsevier. (c) MEGs involved with metal–air redox reaction: Self‐powered humidity sensor based on PSS/PVA/LiCl film (left). Copyrights 2024, Elsevier. Reproduced with permission [34] Moisture absorption‐evaporation cycling fabric MEG (right). Reproduced with permission [35]. Copyright 2024, Science Advances, under a Creative Commons CC BY‐NC 4.0 license. (d) Hydrogel‐based synergistic MEGs: an electrical EDL synergized hydrogel and a supramolecular hydrogel. Reproduced with permission [9, 27]. Copyright 2025, Cell Press and copyright 2024, Springer Nature, under the terms of Creative Commons CC BY 4.0 license.

Optimizing the pathways of ion generation, transmission, and interception processes is a critical approach to enhancing power output performance in moisture‐enabled nanogenerators. Research has centered on modifying the interception layer and nanochannel networks to improve moisture‐electric transition. Feng et al. proposed a synergistic nanoarchitectonics strategy to fabricate MEG through poly(3,4‐ethylenedioxythiophene) (PEDOT): poly (4‐styrenesulfonic acid) (PSS) interception and assembled PSS: porous polyurethane (PU) active layer (Figure 3a, left). The device exhibited a remarkable conversion capability under different humidity and temperature conditions. A striking observation is that this MEG provided sustained high electrical output under high humidity conditions of RH 90%. The ion‐intercepted PEDOT: PSS nano film improved the transportation of hydrogen ions and subsequently enhanced the output performance [2]. Optimization of the ion diffusion and transmission mechanisms prompted numerous studies to focus on modifying the nanoscale channels. Xing et al. introduced a breathable MEG constructed by electrospinning the hydrophilic PVA/phytic acid (PVA/PA) nanofibre membrane and the hydrophobic polyvinylidene difluoride (PVDF) nanofibre membrane (Figure 3a, middle) with various moisture‐absorption abilities. This MEG replaced the neat PVA membrane with a PVA/PA membrane to increase gaseous moisture capture and facilitate ion dissociation, while the nanofibrous architecture contained numerous nanoscale channels that enabled the tubular moisture permeation to assist the oriental ion diffusion and streaming potential generated by molecule transportation [13]. Parallel results were reported by Zhao et al. who applied a 3D‐structured MXene (Ti_3_C_2_T_x_) aerogel film stacked on a PAM hydrogel as the middle transformation layer between top and bottom electrodes (Figure 3a, right). In the aerogel‐on‐hydrogel MEG, directional moisture evaporation through MXene aerogel nanochannels induces hydrolysis, conferring negative surface charges to the nanochannels. These negatively charged nanochannels selectively restrict negative charge ions while facilitating transport of positively charged ions, thereby generating a streaming potential. Concurrent charge accumulation and ion flow dynamics establish a sustained electric field, enhancing energy conversion efficiency. Their MEG device, based on Cu/MXene+PAM/Cu could ultimately increase open circuit voltage and short circuit current from zero for a single MXene device to around 600 mV and 1200 µA [4].

### 2.2 Heterojunctions

Heterojunction‐based MEGs exhibit high practicality due to their more stable electrical outputs under harsh environmental conditions, as junction potentials are formed at interfaces between dissimilar materials. Chen et al. devised an MEG by systematically integrating various ratios of reduced graphene oxide (r‐GO) into graphene oxide (GO) matrices through an ultrasonic mixing method (Figure 3b, left). Benefiting from its hierarchical nanochannel architecture, GO can facilitate water molecule absorption and directional ion transportation and synergize the streaming potential and proton diffusion potential attributed to its gradient in hydrophilic surface functional groups and proton concentration. Experiments found these MEGs could reach a maximum output power density of 15.8 µW cm^−2^ [5]. In another report, Saikia et al. employed the heterojunction of negatively charged vermiculite clay (VM) and oxidized multi‐walled carbon nanotubes (o‐CNT) membranes to fabricate durable MEGs (Figure 3b, right). Their investigations revealed that the junction potential generated at the o‐CNT‐VM interface was the dominant factor in producing the observed electrical outputs, with minor contributions arising from evaporation‐driven ion transport through the 2D nanofluidic channels in the system [6].

### 2.3 Metal–Air Redox Reactions

Except for intelligently utilizing nanostructures and heterogeneous structures, researchers have also employed metal–air redox reactions to accelerate the carrier diffusion rate by setting up metal electrodes, thus increasing the output power. The intentional integration of redox‐active metal electrodes used as supplementary ionic carriers has been developed as one of the key techniques under this concept. Liu et al. developed an asymmetrical structure for their flexible moisture‐enabled electricity generator, which was composed of a bilayer of polyanion (poly(diallyl‐dimethylammonium chloride), PDDA) and polycation (PSS). The excellent ion reserve ability of PDDA and PSS improved the potential difference generated by the dielectric polymer bilayer. To achieve high power output, the incorporation of a metal–air redox reaction was integrated into their system to reduce positive oxygen and promote electron transfer at the electrode interface [1]. Zhang et al. worked on a self‐powered humidity sensor using a LiCl doped PSS:PVA film as electrolyte, paired with Cu and Al electrodes (Figure 3c, left) [34]. The H+ ions in the electrolyte obtain electrons at the positive metal electrode by redox reactions, and the Li+ in the electrolyte breaks the hydrogen bonds of polymers, constructing rapid ion transport as well as increasing ionic conductivity. Similar fabrication of coupling moisture‐electric generation with redox reactions to provide metal cations was explored by Hu and colleagues [35]. In that work, a multilayer asymmetric fibrous structure was built to integrate a moisture absorption layer composed of nonwoven cellulose impregnated with LiCl, with a moisture transport layer of PAN nanofibres and an evaporation layer of poly(vinylidene fluoride‐trifluoroethylene) P(VDF‐TrFE) nanofibres. Through the fibrous assembly, directional moisture transfer was realized, promoting more abundant water adsorption from humid air (Figure 3c, right). Metal cations were supplied continuously through the redox reaction near the electrodes and then driven by the water cycle to move directionally. This, in combination with the charge separation, led to higher electrical outputs. More importantly, these researchers sewed 500 fabric MEGs onto a tent and made practical large‐scale portable energy devices to direct power a mobile phone in outdoor environments.

### 2.4 Hydrogel‐Based MEGs

Chemically treated hydrogels are strategically incorporated into the ionic transportation layers of hydrogel‐based MEGs, where uneven potential fields are induced by selective ion adsorption to improve directional charge migration and energy harvesting. Yan et al. utilized the EDL effect at their hydrogel‐electrode interface and combined it with moisture‐driven ion migration to fabricate high‐performance hydrogel‐based MEGs. Their fabrication utilized asymmetric lithium (Li)‐ion‐doped multi‐walled carbon nanotube (MWCNT) electrodes and a poly(acrylamide‐*co*‐acrylic acid) (P(AM‐co‐AA)) hydrogel to fabricate the EDL synergistic MEG (Figure 3d, left). They used various methodologies, including finite element simulation and first‐principles calculations, to verify that hydrogel‐electrode double layers can function as a booster of power output with environmental tolerance [27]. In another research, Yang et al. introduced an environmentally friendly MEG fabricated based on a supramolecular hydrogel. They applied a PVA‐sodium alginate‐based supramolecular hydrogel (Figure 3d, right) to enhance current generation, by rapid moisture absorption and relatively slow diffusion of ion water clusters. This slow diffusion of ions enables the trapping of water nearby to maintain a water gradient, which benefits continuous electric output [9]. Their work also made advances in employing eco‐friendly materials, aligning with the green and sustainable target toward carbon neutrality.

### 2.5 Hybrid Complex MEGs

**FIGURE 4:**
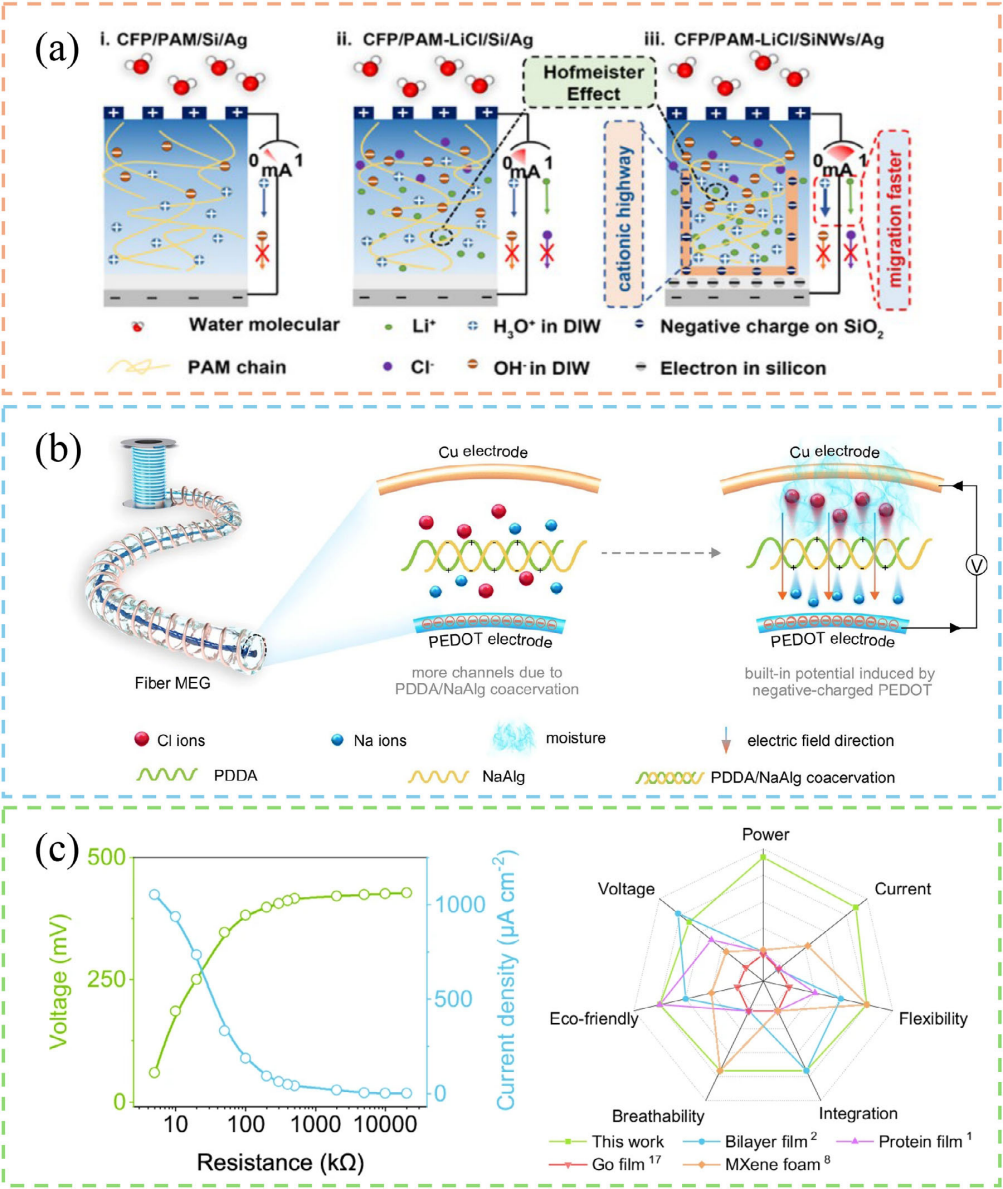
Examples of MEG based on hybrid materials/ structures. (a) Working principle of a hybrid organic‐inorganic MEG. Reproduced with permission [37]. Copyright 2024, RSC Publishing, under the terms of Marketplace permission, general terms and conditions. (b) The fabrication of a coiled triple‐layered MEG with a PEDOT core and a PDDA‐NaAlg shell. (c) Output performance (left) and radar chart of this MEG in comparison with other MEGs (right). Reproduced with permission [10]. Copyright 2024, Springer Nature, under the terms of Creative Commons CC BY 4.0 license.

Hybrid designs have been adopted to overcome the limitations of single‐material or single‐mechanism MEGs. This approach allows for synergistic effects, such as improved moisture absorption, ion transport, and energy conversion, leading to higher power output and greater stability [36]. Duan et al. made an efficient organic/inorganic hybrid MEG via real‐time synthesis of hygroscopic PAM‐based hydrogels on silicon nanowire arrays [37]. Their MEG achieved extraordinary output performance by the synergistic effects of its hybrid design. The hygroscopic PAM ionic hydrogels elevated water absorption and ion dissociation ability, the plentiful cation‐selective nanochannels, and the ion‐induced Hofmeister effect [38] increased the ion migration rate. As a result, the device yielded a record high open‐circuit voltage of 1.28 V at RH 60% and 35°C, and the MEG maintained 60% of the maximum performance even after 800 h of continuous operation. Zan et al. developed a complex coacervation system based on a coiled triple‐layer core‐shell structure, comprising a PEDOT core and a shell coacervate composed of PDDA and sodium alginate (NaAlg), and an outer electrode made of copper threads that enclosed the entire gel fibre (Figure 4b) [10]. They conducted various experiments under different test conditions for a comprehensive investigation of the coacervate gel structures. The statistical analysis of void distribution, free volume fraction, and mean square displacement indicated NaAlg can perform a greater gap prevalence when combined with PDDA polymer. The complex coacervation of two oppositely charged polyelectrolytes created more mobile carriers and free volume, while the PEDOT core promoted carrier diffusion through its surface charges. Other experimental data demonstrated that the PEDOT@PDDA/NaAlg coacervate gel fibre exhibited notable electrical outputs, including high output voltage of approximately 0.8 V, current density of 1.1 mA cm^−2^, and a power density of 184 µW cm^−2^, while maintaining exceptional mechanical pliability (Figure 4c).

The standard evaluation criteria of various construction strategies of MEGs usually encompass output power intensity, humidity dependence, and stability. Table 1 compares the representative MEG configurations and provides each optimal application. The hybrid type and the hydrogel‐based systems currently exhibit the best performance on both power density and long‐term operational stability, whereas nanochannel and heterojunction architectures offer superior humidity sensitivity that can benefit real‐time humidity/respiration sensing and environmental energy harvesters. Metal–air redox configurations provide a convenient route for high transient outputs but face durability issues due to electrode oxidation, therefore the ideal application scenarios are limited to outdoor low‐power generators. Overall, the choice of design strategy should balance output performance, environmental adaptability, and scalability according to the intended application fields.

**TABLE 1:** Comparative evaluation of MEG construction strategies.

Strategies	Typical output power density	Humidity dependence	Stability (Cycles/Duration)	Optimal applications
Nanochannel‐optimized membranes	Moderate	High	Moderate degradation	Real‐time humidity sensors
Heterojunction structures	Low	Moderate	Stable	Environmental energy harvesters
Metal–air redox MEGs	High	Moderate‐High	Limitations in long‐term use	Flexible wearables, any low‐power generators
Hydrogel‐based MEGs	High	Stable over wide RH levels	Excellent	Flexible and biocompatible devices
Hybrid complex MEGs	Maximum	Moderate	Stable	Multifunctional or hybrid energy systems

### 2.6 Environmental Implications of Nanomaterial Synthesis Routes

Environmental implications are inevitable topics for nanomaterials employed in fabricating MEGs. Though these nanostructured materials can significantly improve MEG performance, many commonly used synthesis routes, such as oxidized graphene derivatives and solution processing of MXenes can cause non‐negligible environmental implications. Conventional nanocarbon and MXene syntheses depend on strong acids, high energy consumption, and expensive precursor materials, which offset the sustainability benefits of using moisture harvesters. For instance, conventional GO/rGO synthesis requires concentrated acids H_2_SO_4_ and HNO_3_ or oxidants KMnO_4_, generating significant chemical waste and having high energy consumption [39, 40]. Lifecycle assessment (LCA) studies further show that scaled GO production is dominated by electricity input, reagent consumption, and the required advanced wastewater treatment techniques [41]. MXene fabrication strongly relies on harsh fluoride‐containing etchants such as LiF/HCl or HF, which raise environmental concerns regarding toxicity and uncontrollable industrial pollution that causes challenges in waste management and worker safety induced by solid‐state reaction at high temperature [42].

Recent developments increasingly emphasize the shift toward greener, lower‐cost, and safer synthesis pathways. Naturally abundant minerals such as vermiculite, montmorillonite, and kaolinite can offer environmentally friendly alternatives. For greener MXenes by environment‐friendly synthesis, Amani et al. listed several methods, including low‐temperature phase synthesis, molten‐salt‐assisted etching, and halogen‐mediated etching that can reduce the reliance on strong acids and high‐temperature furnaces [42]. Supramolecular hydrogels and water‐based polymer networks processed at room temperature demonstrate high ionic conductivity and structural tunability without relying on hazardous reagents [9]. In parallel, solvent‐free electrospinning, low‐temperature hydrothermal methods [43] and aqueous ultrasonication routes [44] have also been introduced to further suppress the environmental footprint of nanomaterial fabrication.

## 3 Mechanisms of Moisture‐Enabled Electric Generation

**FIGURE 5:**
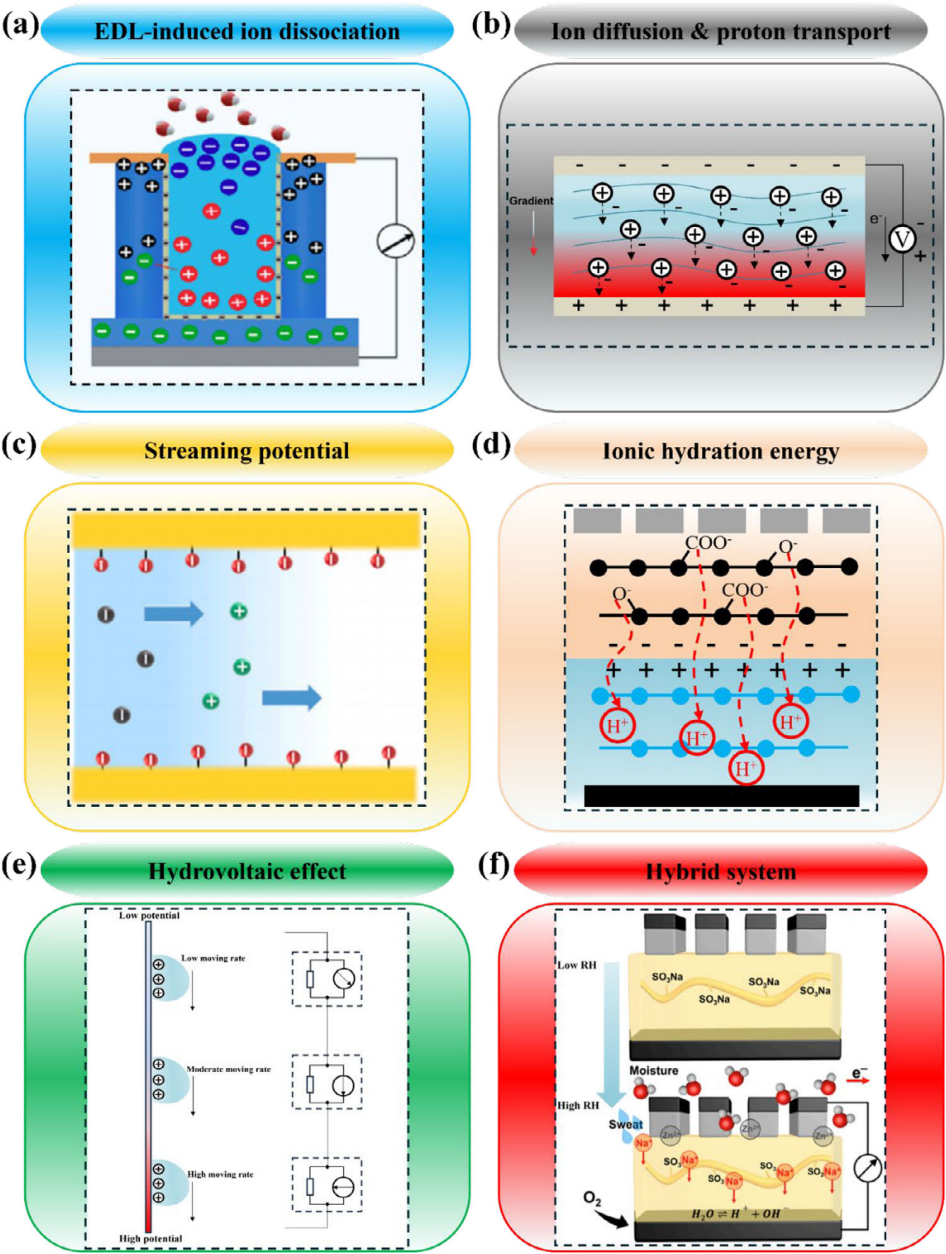
Basic moisture‐to‐electric mechanisms of MEGs. (a) EDL‐induced ion dissociation. Reproduced with permission [15]. Copyright 2023, Elsevier. (b) Ion diffusion and proton transport. (c) Streaming potential. Reproduced with permission [49]. Copyright 2024, Elsevier. (d) Ionic hydration energy. (e) Moisture‐enabled hydrovoltaic effect. (f) Hybrid system and its internal ion motions. Reproduced with permission [16]. Copyright 2024, Elsevier.

MEGs generate electricity through interactions between water molecules and hygroscopic, ionically active materials, with performance determined by both material properties and environmental conditions. Moisture gradients act as the primary driving force for ion migration; when humidity gradients are small or uniform, the achievable potential difference is limited. Temperature further influences MEGs’ operation by regulating water adsorption and desorption behavior and ionic mobility, directly affecting charge transport efficiency. At the material level, ionic conductivity sets the upper limit for current generation and determines the stability of interfacial charge separation, indicating that surface functionalization alone cannot overcome charge transport constraints. Within these physical limits, considerations of utilizing mechanisms to mediate moisture–electric conversion have become an exploratory research field. EDL theory is stated as ionic rearrangements around charged surfaces in an electrolyte to form a potential difference between the electrode and the electrolyte (Figure 5a), which attracts ions to move from the electrolyte toward the electrode's surface [45]. Ion diffusion occurs when the functional groups of the material interact with water and release free ions (Figure 5b). These ions then move under a concentration gradient, forming one of the core mechanisms of producing electricity [46]. The streaming potential is generated by moisture absorbed in a porous or nano‐structured material, when the corresponding ionic flow creates charge separation along the direction of the flow and results in an electric potential difference (Figure 5c). Ionic hydration structures refer to the arrangement of water molecules around an ion in solution. Driven by electrostatic interactions, water molecules are connected to each other forming a hydration shell to maximize the attraction to ion's charge (Figure 5d) [47]. The hydrovoltaic effect is a phenomenon where electricity is generated through the direct interaction of nanomaterials with water in motion: flowing, waving, dropping, and evaporating water. Hydrovoltaic effect can result in electricity generation through the interaction between nanostructured materials with water in different dynamic forms, such as flowing, waving, dropping water, and water in evaporation (Figure 5e). This mechanism potentially improves the water harvesting efficiency in comparison with conventional harvesting of kinetic energy [48]. Hybrid systems applied in MEGs mostly synergize with another energy conversion mechanism (Figure 5f), for example, triboelectric generation, to significantly increase electrical outputs. Such combinational mechanisms have expanded to a larger scale, arrayed power generation systems. Current investigations focus on several strategies to enhance moisture‐electric generation through the utilization of these mechanisms. The following sections listed some of the examples.

### 3.1 EDL‐Synergized Promotions in Streaming Potential

**FIGURE 6:**
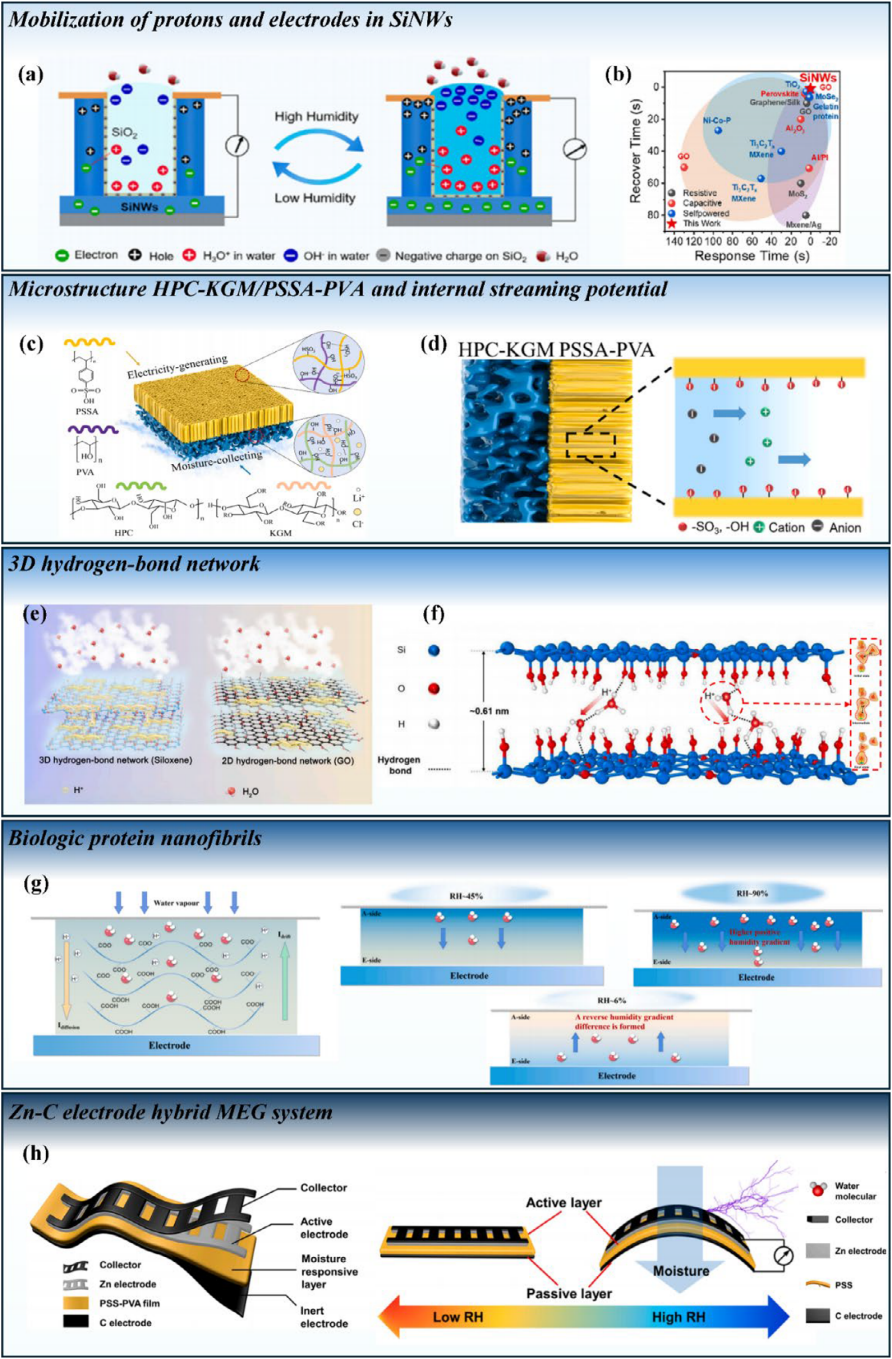
Examples of developing MEGs through different mechanisms. Mobilization of protons and electrodes in SiNWs: (a) The mechanism of ion dissociation and diffusion inside SiNWs. (b) Its response performance compared to previously reported humidity sensors. Reproduced with permission [15]. Copyright 2023, Elsevier. Streaming potential enhanced by microstructure of the HPC‐KGM/PSSA‐PVA aerogel: (c) Composition. (d) Its mechanism of generating streaming potential. Reproduced with permission [49]. Copyright 2024, Elsevier. 3D hydrogen‐bond network: (e) Comparison of the 3D hydrogen‐bond network of siloxene and the 2D hydrogen‐bond network of graphene oxide. (f) Process of forming interconnected bridges for proton hopping, from initial to final states. Reproduced with permission [29]. Copyright 2024, Elsevier. Biologic protein nanofibres: (g) moisture‐enabled hydrovoltaic energy generation. Reproduced with permission [21]. Copyright 2022, Elsevier. Hybrid MEG system: (h) Zn‐C electrode pair with PSS‐PVA hybrid MEG and its electricity generation process. Reproduced with permission [16]. Copyright 2024, Elsevier.

An electric double layer forms at the interface between a solid (can be electrode or hydrogel) and a liquid (like water in the moist air). EDL consists of two oppositely charged regions: a fixed layer of ions adsorbed to the solid surface and a diffuse layer of counterions (ions with the opposite charge) that extends into the liquid. When a fluid flows along the solid‐liquid interface, it can drag ions within the diffuse layer of the EDL, creating a streaming potential. Recent advances demonstrate that nano‐confined EDL environments can take advantage of the properties of the EDL and streaming potential to efficiently extract electrical energy from moisture. By carefully designing the hydrogel‐electrode interface and controlling the humidity gradient, researchers can optimize the performance of MEGs. Song et al. employed super‐hydrophilic silicon nanowires (SiNWs) that attract water on surfaces to simulate the electric double layer and generate a streaming potential [15]. A small fraction of the surface‐accumulated water molecules dissociates into hydronium ions (H_3_O^+^) and hydroxide ions (OH^−^) to build an electric double layer at the interface. The accumulated negative charges create a long‐term Debye shielding effect that compels the confined protons transiting in the nanochannels. This movement simultaneously forms a streaming potential gradient along the double‐layered interspace. As moisture infiltrates the nanochannels, it triggers a screening effect induced by the rising protons and the mobilization of electrons within the nanochannels. The mobilized electrons subsequently diffuse toward the anode with associated hole generation in the cathode (Figure 6a). Attributed to the large surface area of the nanowires and the superior electronic characteristics of semiconductor silicon, this MEG achieved superior outputs (current density of 37 nA cm^−2^ and output voltage of 258.88 mV) compared with conventional carbon‐based MEGs as illustrated in Figure 6b) upon exposure to moisture and exhibited remarkable humidity sensitivity. Yan et al. built their MEGs from multiwall carbon nanotube electrodes and a poly(acrylamide‐*co*‐acrylic acid) hydrogel [27]. The hygroscopic hydrogel and the LiCl doped electrode created a strong EDL and formed a humidity gradient. This MEG exhibited high electrical power output (2.3 µW·cm^2^) and high environmental resistance (0.8 V when RH <30%). Chen et al. also employed similar concepts inspired by arid‐adapted plants to construct a 3D architecture MEG with enlarged hygroscopic surface area, asymmetric directional moisture transport, a strong streaming potential, and continuous spontaneous discharge [50].

### 3.2 Ion Dissociation and Ion Diffusion

Ion dissociation processes, such as the splitting of water molecules into hydrogen ions (H^+^) and hydroxide ions (OH^−^) from ionized water molecules, combined with efficient ion diffusion pathways, can spark a predominant electrochemical potential gradient through the layer‐by‐layer architecture. Currently, some studies collectively advance the field by establishing synergistic structures, such as vertically aligned aerogel channels, ionized hygroscopic polymer pairings and GO‐based heterojunctions. Zhao et al. designed a vertical aerogel‐on‐water‐collecting gel (VAWG) MEG through the unique microstructure of an aerogel formed using poly(4‐styrenesulfonic acid) and poly(vinyl alcohol) (PSSA‐PVA), where the pores of the aerogel were vertically aligned and oriented along the water evaporation direction (Figure 6c) [49]. The integration of a hygroscopic polymer aerogel (hydroxypropyl cellulose/konjac glucomannan/LiCl particles, HPC‐KGM) and an ionized polymer aerogel played a critical role in external water collection and ion transportation. The process of generating streaming potential in this MEG is demonstrated in Figure 6d. The device's high energy‐harvesting performance stems from two synergistic mechanisms: (1) directional moisture transport which utilizes vertical pore alignment in the PSSA‐PVA aerogel to guide water molecules and creates a unidirectional ion diffusion pathway, and (2) a sustained ion diffusion process in the HPC‐KGM aerogel, which initiates the continuous water supply to enable prolonged proton migration and charge separation within the PSSA‐PVA matrix. These findings demonstrated that the streaming potential and ion diffusion process, as fundamental mechanisms of MEGs, can be realized in optimized synergistic polymer pairs (like generating ion diffusion between two EDL) and can significantly promote the efficiency of electricity generation of MEGs.

In Chen's devised GO/rGO MEGs, the purpose‐built GO/rGO heterojunctions increased the ion concentration differences due to the hygroscopicity distinctions between GO and rGO, therefore improving the electrical voltage and current outputs [5]. At low humidity levels, the potential difference between the top and bottom electrodes was small due to the limited H+ ions from the environment. As humidity increased, water molecules were rapidly physiosorbed onto the upper surface of the MEG through its porous top Ni electrode and activated the ionization process. This process would ionize partial water molecules through the rich ─OH and ─COOH groups in the GO layer. The remaining water molecules subsequently accumulated on the Ni/GO interface, forming a moisture storage layer, and initiated the downward potential gradient. This gradient drives diffusion‐induced current between electrodes. An optimal GO/rGO ratio of 10/1 lowers the electrical resistance in the hybrid functional layer and improves the current output. After moisture removal, the evaporation initiated by molecules from the Ni/GO interface reservoir drove the mobilized hydrogens to dissipate back to the upper surface of the MEG, regenerating the charge distribution. Facilitated by strong ionization, this flexible MEG showed no noticeable output degradation after 3000 times flexure loading at 2 cm deflection.

### 3.3 3D Hydrogen‐Bond Network

3D hydrogen‐bonding networks are hydrogen‐based intermediate structures that can encourage proton transport. Dynamically reconfigurable hydrogen‐bond network can fundamentally alter proton migration pathways. Wang et al. developed a dynamic 3D hydrogen‐bond network MEG by taking advantage of the corrugated 2D structure of siloxane and its ample oxygen‐containing functional groups [29]. The distinction of the 3D hydrogen‐bond network from siloxene and the 2D hydrogen‐bond network from GO is illustrated in Figure 6e. The innovative 3D network facilitates multidirectional random hopping of dissociated protons—a mechanism distinct from conventional linear diffusion. Differing from the traditional diffusion mechanism, the network can form dense and effective interconnected bridges for proton hopping, significantly assisting the proton migration to overcome the barriers remaining in traditional unidirectional diffusion. The MEG mechanism was explored through two scenarios of proton transformation, i.e., within the plane and proton transformation with the interlayer region with multiple ─OH groups (Figure 6f). The study elucidated two fundamental principles governing dynamic 3D hydrogen‐bond networks, i.e., one is kinetic barrier mitigation, which allows networks to overcome proton migration energy barriers through adaptive structural reconfiguration, minimizing kinetic limitations, and the other is the Grotthuss diffusion mechanism when the dissociated protons hopping along the hydrogen‐bond network [51].

### 3.4 Moisture‐Enabled Hydrovoltaic Effects

Biological materials have emerged as promising candidates for moisture‐enabled hydrovoltaic energy generation due to their inherent biocompatibility and tunable physicochemical properties [52]. The critical advances, particularly focused on protein nanofibrils, which can intrinsically couple water adsorption, ionization, and proton transport to yield unexpected moisture‐electric conversion. In a pioneering study, Liu et al. reported a MEG based on protein nanofibrils extracted from milk β‐lactoglobulin [21]. The device achieved excellent performance (maximum power density of 38.88 µW cm^2^) through synergistic structural and property attributes, including exceptional hydrophilicity, high surface charge density, and nanoscale fibrillar architecture. The energy harvesting mechanism of PN‐MEG is demonstrated in Figure 6g. Water molecules from the humid air diffused through the titanium mesh and reached the nanofibril membrane, before condensing into liquid form and being absorbed onto the hydrophilic amino‐acid side groups. Density functional theory (DFT) calculations revealed that the carboxyl functional groups of amino acid chains in the protein nanofibrils were the active sites for water molecule adsorption and ionization. The energy released through this phase change, combined with the absorption energy to act on hydrophilic functional groups such as –COOH on the nanofibril surfaces. Ionization of water molecules is then initiated and generates free hydrogen ions (H^+^) that subsequently dissociate from their absorption sites, creating a potential difference and an electric field. They also explained the ion diffusion by the osmotic pressure effect [53].

### 3.5 Hybrid MEG Systems

Moisture‐enabled electric generation can also be realized in hybrid systems so moisture could be utilized multifunctionally, including for power generation and actuation to enable human‐machine interaction/sensing. Zhan et al. fabricated their moisture‐triggered hybrid actuator and electric generator (MTAEG) based on the above concept [16]. They employed an active layer of a mixed PSS‐PVA composite membrane containing ion exchange channels to facilitate selective ion transport. A passive carbon‐based electrode functionalized with nanocomponents enhanced electrical conductivity and interfacial contact. An electroactive layer of a Zn anode served as the redox‐active component for electron donation, while a corrosion‐resistant metallic foil served for current collection. The rational synergy between the asymmetric electroactive electrodes and polyelectrolytes with excellent ion conductivity and hygroscopicity led to a maximum bending angle >125° at RH 80 % and generated electric power of maximum density of 11.24 µW cm^−2^ at RH 99 %. The moisture‐triggered mechanism of MTAEG is shown in Figure 6 h.

In hybrid MEG systems, multiple mechanisms can coexist, which are developed to overcome the existing issues and realize notable performance boost. One example is the innovative MEGs based on SiNWs fabricated by Song's team [15]; the superior current density and voltage were triggered by the synergistic effect of EDL interfaces and nanochannel‐induced streaming potential. Other inspiring developments of complementary moisture‐electric energy mechanisms can be found in hybrid MEGs, e.g., Kim et al. merged deformable MEG and TENG technologies into a single cell [17], while Pei et al. developed hybrid system combining a fibre‐based TENG with yarn‐based sweat‐activated batteries to achieve a dry‐wet bimodal power supply [54]. These developments indicate multiple mechanisms can coexist in a single MEG. Various research works also found that the mechanisms can operate cooperatively rather than competitively under optimized conditions, moderate RH levels, room temperature, and periodic moisture cycling [55, 56, 57]. The synergistic effects are mainly represented by continuous voltage provided by the diffusion potential, additional carriers contributed from redox reactions, and streaming effects that enhance directional ion transport. The multi‐mechanistic coupling progressively generates higher power density and improved stability compared to single mechanism MEGs.

## 4 Positioning MEGs in Different Ambient Energy Harvesters

To enable a meaningful comparison with other ambient energy‐harvesting approaches, this section examines various harvesters in terms of power density, scalability, cost considerations, and applications‐specific requirements, relative to MEG systems. Typical energy harvesters such as thermoelectric, triboelectric, and piezoelectric generators, as well as biofuel cells [58], have undergone extensive development over many years. In recent years, newly emerged harvesting technologies based on distinct transduction mechanisms have gained attention, particularly electromagnetic generators (EMGs) and regenerative organic Rankine cycle (RORC) systems. To evaluate these technologies and compare their respective advantages and disadvantages relative to MEGs, representative configurations reported in the literature were selected. These systems were analyzed in terms of output performance, cost–benefit ratio, scalability of fabrication, and additional applications‐specific tradeoffs.

### 4.1 Energy Output

Typical open‐circuit voltages for contact‐mode TENGs fall within 50–500 V, and outputs exceeding 500 V are frequently reported in high‑voltage device designs. However, the influence of renewable materials, including biopolymers and their monomers on TENG performance remains poorly understood [59]. Previous investigations demonstrated a high‐voltage output from chia‑seed–based TENGs (C‑TENGs), which generated an open‐circuit voltage of 502 V and achieved a power density of 290 mW/m^2^ [60]. This representative bio‑TENG successfully overcame the low‑voltage limitation in earlier bio‑TENG designs, which often have only tens of volts or even single‑digit outputs [61, 62]. Nevertheless, the tribo‑negative layer in this device was PTFE, a fluoropolymer widely recognized as non‑bio‑based and non‑renewable. Load‑matching measurements of the C‑TENG yielded a maximum power density of 0.29 W/m^2^. Although this characterization method is more scientifically rigorous than finger tapping, the resulting output remains insufficient for powering applications beyond low‑power demonstrations such as LEDs or calculators [60]. In contrast, newly developed MEGs can utilize lignosulfonate as the active functional material paired with graphite electrodes, enabling a fully bio‑based device configuration. This design is simple, sustainable, and low‑cost, and has achieved a maximum power density of 0.327 W/m^2^ under 98% relative humidity at room temperature [63].

Similarly, only a limited number of studies have explored the use of bio‑based materials in piezoelectric nanogenerators (PENGs). Recent work has introduced degradable flexible PENGs fabricated from BaTiO_3_ and polylactic acid via spin‑coating. In the 2023 report by Zhao et al., although the energy output was not explicitly stated, calculations based on the effective working area indicate a power density of 0.745 µW/cm^2^, which is significantly lower than those of bio‑based TENGs and MEGs [64]. From a biodegradability perspective, the design is not fully bio‑based, as BaTiO_3_ is an inorganic ceramic, and its synthesis inevitably generates environmental impacts.

A traditional method for utilizing large waste‐heat sources is the organic Rankine cycle (ORC), in which heat is used to vaporize an organic working fluid to drive a turbine and generate electricity. However, the high calibration and maintenance requirements of ORC systems contribute to significant operational costs. In contrast, thermoelectric generators (TEGs) provide a direct route for converting temperature gradients into electrical power [65]. In 2025, a predominantly bio‑based thermoelectric gel material was developed using an all‑wood ionic gel. This system achieved enhanced thermopower through a lignocellulose channel reconstruction strategy within the wood cell structure. As a trade‐off for its strong sustainability, the device delivered an energy density of 68.75 J/m^2^, corresponding to a power density of approximately 9.6 mW/m^2^ [66] that is much lower than the output of MEGs fabricated from chitosan and sodium lignosulfonate, which reported power densities up to 14.8 µW/cm^2^ [67] and demonstrated potential for green industrial energy applications.

R‑ORC, a regenerative variant of the conventional ORC, is designed to convert thermal energy into electricity more efficiently. Importantly, R‑ORC systems can harvest heat from renewable resources such as biomass and solar energy, which played an influential role in advancing ORC technologies toward sustainability. Experimental studies examining the heat‑source loop, ORC loop, and cooling‑water loop have shown that R‑ORC systems can achieve thermal efficiencies approximately 25.5% higher than those of traditional ORC configurations [68]. Although no direct experimental comparison between R‑ORC and MEGs exists, owing to their fundamentally different operating mechanisms. The contributions of R‑ORC to sustainable energy harvesting are noteworthy, and the emerging concept of integrating nanogenerators into ORC architectures to construct hybrid energy systems is drawing increasing research interest.

In 2025, enzymatic sweat biofuel cells (BFCs) were reported to have areal power densities of 1.6 mW/cm^2^. These devices utilize enzyme‑modified electrodes to catalyze lactate oxidation at the anode and oxygen reduction at the air cathode, generating electrical current through an external circuit. By using lactate in human sweat as a biofuel, these BFCs maintain relatively low environmental footprints. Under optimized conditions, their areal power densities can reach 16 W/m^2^ [69], surpassing those of contemporary advanced MEGs. However, BFCs require biological fuels and enzyme‑containing electrodes, whereas MEGs operate without consumable fuels and rely solely on ambient humidity. Thus, the two technologies must be evaluated separately in terms of sustainability and practical applicability. Although sweat‑powered systems are technically feasible for wearable electronics, they are inherently less sustainable than moisture‑electric generators when assessed at the level of real‑world use cases.

Electromagnetic generators (EMGs) operate based on the principles of electromagnetic induction and resistive free‑electron conduction driven by the Lorentz force [70]. A representative high‑output electromagnetic vibration energy harvester has demonstrated that the volumetric power density can reach 4.25 mW cm^−^^3^ under 60 Hz, 1 g RMS vibration conditions [71]. In comparison, an innovative polysaccharide‑based MEG has introduced a promising paradigm for high‑performance, bio‑derived MEGs, achieving an improved volumetric power density of 1291 W m^−^^3^ [72]. However, the energy conversion mechanisms for MEGs lead to distinct advantages, since they harvest energy from ambient moisture or humidity without requiring deliberate or periodic vibration input that is essential for EMG operation.

### 4.2 Levelized Cost of Electricity

Levelized cost of electricity (LCOE) is an evaluation criterion to calculate and analyze the required cost to produce the same unit quantity of electrical energy [73]. It is widely used in defining the benefit‐cost level of energy harvesting technologies. LCOE allows the comparison of different technologies of unequal lifespans, different device sizes, capital costs, and capacities. The criterion neglects the maintenance cost due to the hardness of collecting data in each maintenance cycle.

(1) $$\mathrm{LCOE}=\frac{\mathrm{Sum}\;\mathrm{of}\;\mathrm{Costs}\;\mathrm{over}\;\mathrm{Lifetime}}{\mathrm{Sum}\;\mathrm{of}\;\mathrm{Electrical}\;\mathrm{Energy}\;\mathrm{Produced}}\;$$

Wang et al. compared several energy‑harvesting technologies by calculating their levelized cost of energy (LCOE) and showed that EMGs exhibited the highest LCOE, followed by PENGs, and then TEGs, with values of 278.95, 106.39, and 95.74 USD/kWh, respectively [73]. Earlier economic assessments of TENGs, conducted in 2017 using a comprehensive techno‑economic framework, reported LCOEs ranging from 2.569 to 9.43 US cents/kWh for two TENG modules [74]. These results demonstrate that TENGs have achieved substantial progress toward low‑cost energy harvesting, consistent with the findings summarized by Yao et al., whose index‑based comparison also included the cost performance of BFCs [58]. In a comparative work by Mokhtari et al., three configurations, i.e., basic ORC, ORC with a reheater, and R‑ORC, were evaluated in terms of their LCOEs. The optimized R‑ORC off‑grid configuration delivered the lowest LCOE at 4.52 cents/kWh [75], a value notably close to the prevalent cost range of TENGs. Compared with the above energy‑harvesting technologies, MEGs have attracted attention for their potentially significant cost advantages. Zhu et al. conducted a detailed economic analysis of a whey‑protein‑based MEG, reporting a minimum chemical cost of 17.61 AUD/m^2^ that is approximately 70 times cheaper than commonly used polymer‑based materials [76]. These findings align with Yao et al.’s classification of MEGs as an extremely low‑cost technology [58]. A cost evaluation and LCOE comparison of different energy generators was therefore accomplished in Table 2.

**TABLE 2:** Economic assessment of different energy harvesting technologies.

Type★	MEGs	TEGs	TENGs	PENGs	BFCs	EMGs	R‐ORC
Cost	Extremely low	High	Low	Moderate	Moderate to high	Extremely high	Low
LCOE	MEGs < TENGs < R‐ORC < PENGs < BFCs < TEGs < EMGs						

### 4.3 Technological Maturity, Scalability, and Application‐Specific Trade‐Offs

Beyond absolute analysis of power output and economic assessment, the practical potential of ambient energy harvesters is strongly governed by their technological maturity, scalability, and suitability for specific application scenarios. Situating MEGs within this broader technological ecosystem is therefore essential for a balanced assessment of their strengths and limitations.

TENGs and PENGs represent relatively mature classes of mechanical energy harvesters, with well‐established device architectures, standardized characterization approaches, and demonstrated integration in wearable systems, structural monitoring, and self‐powered sensors [77, 78]. Scalable fabrication strategies, such as textile integration, multilayer stacking, and roll‐to‐roll processing, have been widely reported. However, both TENGs and PENGs fundamentally depend on intermittent mechanical excitation, which limits their operation cycle and time‐averaged power output in static or low‐motion environments. As a result, while high peak power densities are frequently reported, sustained power delivery under realistic operating conditions is often significantly lower [79].

TEGs and ORC systems occupy a more technologically mature position, particularly at medium to large scales. TEGs offer key advantages such as solid‑state operation, mechanical robustness, and long service lifetimes; however, their practical deployment for low‑grade heat recovery remains limited by inherently low conversion efficiencies and the high cost of thermoelectric materials [80]. In contrast, ORC and R‑ORC systems have benefited from decades of thermodynamic refinement and techno‑economic analysis, resulting in well‑established cost trajectories and proven scalability from the kilowatt to megawatt range [81, 82, 83]. Recent studies have further pushed ORC concepts toward effective low‑temperature operation, demonstrating the feasibility of harvesting low‑grade thermal energy through the use of advanced working fluids and multi‑stage expander architectures [84]. Despite these advances, the dependence of ORC systems on turbines, heat exchangers, and complex working‑fluid management inherently confines their deployment to centralized or semi‑centralized settings, rendering them unsuitable for distributed, flexible, or wearable electronic applications.

Against this backdrop, MEGs remain at an earlier stage of technological maturity but address a distinct operational niche. By harvesting energy directly from ambient moisture or humidity gradients, MEGs can operate continuously in environments where mechanical motion or temperature gradients are insufficient, such as indoor spaces, enclosed infrastructures, or 24 h power‐supplied settings [46, 76, 85]. Recent advances have demonstrated scalable fabrication strategies for MEGs, including paper‐based substrates, polymer and hydrogel casting, and textile‐compatible architectures, indicating compatibility with large‐area and low‐temperature manufacturing routes [86, 87, 88]. However, most MEG demonstrations remain at laboratory or pilot scale, and challenges related to long‐term stability under fluctuating humidity, encapsulation, and device‐to‐device reproducibility remain unresolved.

MEGs should not be considered competitors to high‑power or centralized energy‑harvesting technologies; rather, they function as complementary systems designed for low‑power, distributed, and maintenance‑free applications. Their distinctive operating mechanism, combined with emerging scalable fabrication strategies such as paper‑based biofilm production, hydrogel casting, knitted and modular wiring architectures, and other low infrastructure, easily reproducible manufacturing methods, provides multiple pathways for systematic scaling. These evolving fabrication approaches position MEGs as promising candidates for powering self‑sustained sensors, IoT nodes, and embedded building‑scale electronics. At the same time, their development trajectory underscores the need to address current limitations, offering clear opportunities for future research aimed at enhancing durability, environmental stability, and integration versatility.

## 5 Enhancing Energy Harvesting Performance of MEGs

MEGs are advantageous due to their ability to harvest energy from moisture. However, current studies have shown that the power density of MEGs is constrained by the material‐limited ion mobility as described in Section 4. At the same time, high internal resistance is an architecture‐related issue that can worsen the constraints of output performance. For most early MEGs, electric field degrades within tens of seconds because of the limited coverage of functional groups and fast proton transport [89]. Stability problems are mainly found after repeated dehydration and structural failure, which can cause some ions not be able to return to the initial position or lead to increased ionic resistance [90]. The scalability challenge is a derivative of low unit power in the system. Most MEG designs can only generate a low current density of below 10 µA cm^−^^2^, which cannot meet the requirement in a scalable system and has the bottleneck of assembling many units with the current techniques. Based on these facts, it is critical to enhance the output performance of MEGs by various approaches, including material innovation and integration with other energy harvesting methods. In this section, the advanced MEGs are introduced and analyzed according to two main categories, i.e., intrinsic enhancement and hybrid enhancement. Six concepts of both types of advances through material‐driven, structure‐driven, and multi‐mechanism driven techniques are discussed.

### 5.1 Film Structures

**FIGURE 7:**
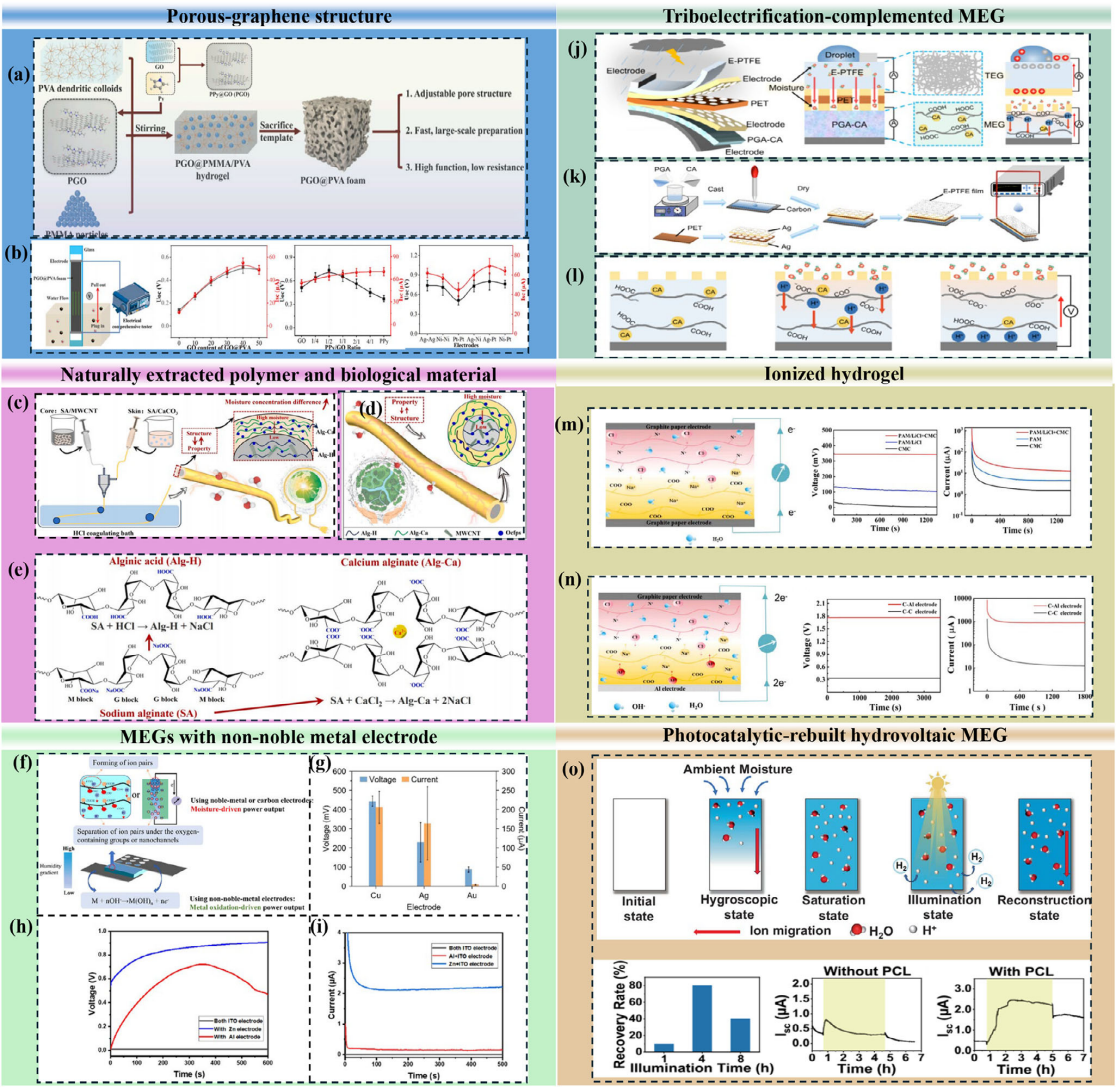
Different methodologies to improve the moisture‐electric harvesting capacity. Creating innovative porous‐graphene structures, (a) porous PGO@PMMA/PVA hydrogel. (b) Its output performance under different content and different electrode pairs. Reproduced with permission [91]. Copyright 2023, Elsevier. Naturally extracted polymers and biological materials. (c, d) Design concept and spinning process of Alg/MWCNT coaxial fibres. (e) Chemical reactions and structures of Alg‐H and Alg‐Ca. Reproduced with permission [95]. Copyright 2024, Elsevier. MEGs with non‐metal electrode. (f) Mechanism comparison between using noble‐metal or carbon electrodes and using non‐noble metal electrodes. Reproduced with permission [97]. Copyright 2023, ACS Publications. g) Output voltage and current of MXene/PAM MEG with different electrodes composed of Cu, Ag, and Au. Reproduced with permission [4]. Copyright 2023, ACS Publications. (h) Voltage outputs with the replacement of the bottom electrodes by ITO, Al, and Zn. (i) Current outputs with the replacements of the bottom electrodes by ITO, Al, and Zn. Reproduced with permission [99]. Copyright 2022, RSC Publishing, accepted by Marketplace Permissions General Terms and Conditions and any applicable Publisher Terms and Conditions. Triboelectrification‐complemented MEG. (j) Structure and functional regions of the hybrid nanogenerator. (k) Preparation process of the porous PTFE film. (l) Working mechanism of the moisture‐enabled functional layer. Reproduced with permission [18]. Copyright 2024, ACS Publications. Ionic hydrogel moisture electricity nanogenerator. (m) Principle when carbon–carbon electrode was adopted, and voltage and current comparison graphs of the ionic hydrogel MEG. (n) Alternative performance when the carbon‐aluminium electrode is employed. Reproduced with permission [22]. Copyright 2025, Elsevier. Photocatalytic‐hydrovoltaic MEG. (o) Mechanism and verification of photocatalytic effect on reconstructing ion concentration gradient. Reproduced with permission [19]. Copyright 2025, Springer Nature, under the terms of the Creative Commons CC BY 4.0 license.

Early design of films for MEGs was under the implicit assumption that maximizing surface area alone would proportionally increase electrical output. Recent film‐structure innovations challenge this assumption by demonstrating that the spatial organization of moisture, ions, and electric fields is more critical than surface area itself. Zhang et al. prepared polypyrrole (PPy)‐modified GO‐filled PVA foam (using polymethyl methacrylate (PMMA) particles as a template for porous structure formation) (as shown in Figure 7a). Rather than individually increasing adsorption sites, the interconnected pores establish distributed micro‐reservoirs of moisture, enabling sustained ion dissociation throughout the film thickness instead of at a single interface (Figure 7b) [91]. This transforms the MEG from a surface‐driven device into a bulk‐driven electrochemical system, mitigating rapid field decay. Similarly, paper‐based and biofilm‐derived MEGs introduce a new design paradigm of directional moisture flux control [92]. Janus paper layers do not simply absorb water; they rectify moisture transport, creating a persistent internal gradient that continuously drives ion migration. This reframes film design from “humidity‐responsive” to “gradient‐maintaining”, which is essential for long‐duration operation.

Beyond porous structure, more sophisticated film structures, including homogeneous, gradient or heterogenous functional films, have emerged for optimizing MEG outputs. Homogeneous MEG films can ensure consistent ion conduction pathways through uniformly distributed functional groups. The development of 3D, self‐sustained MEG successfully achieved efficient and persistent power generation [50]. The wood‐based ionic hydrogel was further treated for asymmetric wettability and to realize directional moisture transport, a volumetric streaming potential, and sustained spontaneous discharge. By contrast, gradient structures introduce spatial asymmetry in moisture adsorption and charge carrier concentration, generating stronger internal driving forces for ion migration. Huang et al. developed a double‐gradient structured hydrogel to synergize the hydrophilicity gradient and the ion concentration gradient in their developed MEG [93]. This MEG exploited asymmetric hydrophilicity to create a water gradient and sustain electric output. They also promoted their design in scalable MEGs to directly power commercial electronic devices without the requirements of supplementary rectifiers or capacitors, which can be marked as a significant breakout in scalable MEGs. Meanwhile, heterogeneous structures are often inspired by bioresource, containing distinct charge densities or ionic mobilities to generate interfacial electric fields. A heterogeneous bilayer system of cellulose nanofibers can have one layer functionalized with carboxylate groups and the other layer with quaternary ammonium moieties [94]. The asymmetric ion‐distribution enabled stable moisture absorption and sustained ion diffusion, which allowed the MEG to operate even under extreme humidity fluctuations without collapsing the equilibrium state.

### 5.2 Biomaterials for MEG

Biomaterials offer a sustainable and biocompatible alternative to traditional materials for energy harvesting, particularly in applications like wearable electronics and self‐powered sensors. However, their practical application is constrained by low power density. Recent progress in MEGs has transferred from simple material replacement toward mechanism‐driven design strategies that exploit spatial asymmetry and molecular‐level ion–water interactions to amplify electrokinetic output [92].

Zhang et al. utilized alginate (Alg) and MWCNT coaxial fibres to create moisture adsorption gradients in the radial direction for improving spontaneous electrical generation [95]. By converting sodium alginate into calcium alginate (Alg‐Ca) and alginic acid (Alg‐H), and incorporating conductive MWCNTs into the Alg‐H core, sustained charge separation was achieved through coupled gradients in hydration capacity and oxygen‐containing functional groups relative to the Alg‐Ca shell adjacent to skin (Figure 7c–e). In addition, the concentration difference of Alg‐Ca along the radial direction also contributed to superior electrical output and continuous power generation. Liu et al. further advanced MEG design by linking macroscopic electrical output to molecular‐scale water adsorption and ionization mechanisms in protein nanofibrils (PNs) derived from milk β‐lactoglobulin [21]. DFT calculations [96] identified carboxyl groups on amino acid chains as dominant adsorption and ionization sites, with adsorption energy quantified as:

(2) $$E_{ads}=E_{total}-\left(E_{Aacf}+E_{H_{2}O}\right)$$

where *E_total_* is the total energy adsorbed by the system, *E_Aacf_* is the energy of the amino acid chain fragment, and $E_{H_{2}O}$ is the energy of free water molecules. Kelvin probe force microscopy revealed humidity‐dependent increases in surface potential, directly correlating with output voltage and confirming enhanced ion dissociation at higher water uptake. Comparative studies showed milk protein films to be more hydrophilic than ovalbumin and silk proteins, enabling greater atmospheric water absorption and more efficient internal charge transport. Together, these findings establish protein‐based MEGs as chemically programmable systems, whose performance can be rationally engineered through functional group chemistry rather than empirical material selection [21].

### 5.3 Low‐Cost Metals as Electrodes for MEGs

Non‐noble metals are increasingly employed as electrodes in MEGs not only for economic reasons, but because they introduce electrochemically active interfaces that fundamentally redefine the energy‐conversion methodology. Under humid conditions, exposed non‐noble metals have spontaneous redox reactions, while permeated moisture between electrodes effectively forms a metal–air electrochemical system. In this configuration, anodic oxidation and cathodic reduction drive ion exchange and electron transport, leading to substantially higher output compared with noble‐metal or carbon‐based electrodes (Figure 7f) [97]. These findings mark a conceptual transition from treating electrodes as passive charge collectors to recognizing them as active contributors to moisture‐induced power generation.

Electrode pairing has consequently emerged as a critical design parameter determining internal potential gradients and redox kinetics. In MXene/PAM‐based MEGs, Zhao et al. showed that Cu electrodes generated open‐circuit voltages of almost 450 mV and short‐circuit currents exceeding 200 µA, far surpassing devices employing Au or Ag electrodes (Figure 7 g) [4]. This is because, in addition to power generation arising from the interaction of MXene and water, the redox reaction also contributes to power generation. When the Cu cathode and anode are connected to the external circuit, the released Cu^2+^ and electrons induce migration of hydroxides and cause an increase in internal potential gradient.

Whether silver electrodes undergo redox reactions under humid conditions remains an important question. Silver is typically regarded as relatively inert. However, Ag can oxidize to Ag_2_O or Ag^+^ species in the presence of moisture, oxygen, and specific electrolytes. When such reactions occur in MEGs, they could contribute significantly to the electrical output. Yang et al. demonstrated that the redox reactions occurring at the silver electrode surface can substantially contribute to output voltage in a fully printable MEG. They explained the significant rise in discharge capacity for cells with silver electrodes through the steady conversion of ion migration to electron transportation via continuous redox reactions. The cyclic voltammetry (CV) curve of the cell using gold current collectors showed no obvious redox reactions; however, the cell with silver electrodes displayed clear redox peaks and high reaction current when it was charged and discharged. This indicates the participation of silver in the redox reactions [98].

Beyond individual materials, capacitor‐inspired MEGs have highlighted the synergistic role of electrode–electrolyte interactions in spontaneous charge amplification. Sun et al. reported that Zn–ITO electrode pairs generated voltages approaching 1 V and currents up to 2.5 mA, even without an added electrolyte layer, significantly outperforming Al–ITO systems (Figure 7 h,i). The MEG with Zn‐ITO electrodes generated remarkable voltage and current outputs of around 1 V and 2.5 mA, which surpassed 2 times in voltage and 250 times in current of that with Al‐ITO electrodes. Additionally, Zn played another vital role in this reaction since it formed an EDL layer when absorbing hydroxide ions from the moisture, which significantly accelerated ion diffusion, synergized with absorbed negatively charged ions from the electrolyte [99]. Other studies focused on the PAN/PSSA fabrication replacing ITO by Al as the top electrode to enhance voltage output [100], and the wood sponge‐LiCl construction of MEG with different electrodes of Pt, Au, Zn, Fe, graphite, and CH‐8 [101], establishing a new electrode‐centric design paradigm for MEGs, in which moisture‐induced electrochemical activity at redox‐active metal interfaces is deliberately harnessed to overcome power‐density limitations. Future advances are therefore expected to arise from rational coupling of electrode redox chemistry, interfacial ion transport, and hygroscopic functional layers, rather than from material substitution alone.

### 5.4 Energy‐Complemented MEGs

Most MEGs suffer from low electrical output due to their reliance on a single, humidity‐driven energy conversion. Recent studies adopted energy‐complemented architectures to overcome this limitation. Deliberately integrating multiple transduction mechanisms within one unified device boosts synergistic power generation to achieve additive performance gains. This methodology reformed traditional MEGs from optimizing isolated MEGs toward multi‐physics co‐harvesting platforms.

A flexible and highly efficient triboelectrification‐complemented moisture electric nanogenerator based on the water‐absorbent citric acid (CA) mediated polyglutamic acid (PGA) hydrogel (as moisture‐electricity generating functional layer) and the porous electret expanded polytetrafluoroethylene (PTFE) (as triboelectric layer to capture impact energy from water droplets) was introduced by Li and colleagues (Figure 7j–l) [18]. The porous PTFE tribolayer plays dual functions, i.e., with one of them driving efficient triboelectrification as water droplets impact the solid material due to its high charge storage property, and the other function facilitating moisture transport through the micro‐sized holes into the hydrogel layer for moisture electricity generation. The hygroscopic hydrogel layer has strong water adsorption capability, ascribed to the abundant carboxylic acid groups, which leads to proton dissociation. In combination with the asymmetric structure for moisture transport inherent in the device construction, the resulting selective ion movement can cause efficient separation of positive and negative charges and generate high electrical outputs. Importantly, the triboelectric process does not merely supplement the MEG output; instead, it modulates interfacial charge density and local electric fields, reinforcing the moisture‐induced electrokinetic process. The work illustrates energy‐complemented MEGs as hybrid transduction systems, in which mechanically induced surface charging and chemically driven ion transport are functionally coupled. These architectures challenge the conventional view of MEGs as single‐source harvesters and suggest that advanced high‐output MEGs will benefit from intentional co‐design of multi‐energy incentive mode, shared charge reservoirs, and interfacial coupling mechanisms, replacing incremental optimization of individual components.

### 5.5 Ionic Hydrogels

Ionic hydrogels have emerged as a particularly powerful platform for MEGs because they combine continuous water uptake, high ionic mobility, and structural adaptability within a single material system. Conceptually, they change MEG design away from solid or gas interfacial charging toward bulk, volumetric ion transport charged by moisture and polymers coupling, hence generating higher charge densities and faster responses under humid environments.

Cao and colleagues’ recent research focused on developing new MEGs based on ionized hydrogels [22]. Cationic PAM (incorporated with LiCl) and anionic carboxymethyl cellulose (CMC) were stacked together to construct the ionic hydrogel. The PAM hydrogel possesses hydrophilic functional groups that dissociate and release anions upon exposure to moisture, leading to positive electrical properties. The CMC hydrogel contains functional groups like hydroxyl and ester groups, which can dissociate and release cations upon moisture contact, causing negative electrical properties of the polymer. The MEG adopted both the graphite electrodes (Figure 7m) or one graphite electrode with the opposite Al electrode (Figure 7n). When the bottom electrode is replaced by the Al electrode, the ionic concentration gradient is enlarged because the asymmetric electrodes brought new cations and anions by electrochemical reactions. As a result, the output performance was significantly enhanced, reaching a peak power density of 113 µW cm^−2^.

Recent advances have further demonstrated the potential of ionic hydrogels in building high‐performance MEGs. Ying et al. reported a high‐power ionic hydrogel MEG, which integrated rGO nanosheets and LiCl into a PAM hydrogel [57]. In their design, rGO nanochannels play a synergistic function with LiCl to realize enhanced ion transport and hygroscopicity in the PAM matrix. This design highlights a key conceptual advance: decoupling ionic conductivity from bulk swelling by introducing nanoscale transport networks, enabling simultaneous improvements in power density and mechanical stability. Pi et al. conducted an inspiring work focused on aging treatment of hydrogel active materials to study their ionic conductivity and water absorption [102]. Through using the direct laser writing carbonization method, they found that the ageing treatment and corrosion reaction can determine ionic conductivity, water uptake ability, and power performance of MEGs. Additionally, the enhanced polymer‐engineering strategies, for example, dual‐network hydrogels, have shown to further improve moisture‐absorption and stability in ionic hydrogel‐based MEGs [103]. Collectively, these studies establish ionic hydrogels as active, reconfigurable ionic conductors, rather than passive hygroscopic films. Superior performance can be resulted from ion‐moisture interactions and interactions within various electrochemical boundaries.

### 5.6 Photocatalysis‐Assisted Hydrovoltaic MEGs

The output performance of MEGs has been continuously improved in the past 10 years, increasing from the first reported MEG in 2015 by Qu's group, whose power density was 18.4 nW/cm^2^ [104] to a current high‐output MEG with 12 µW cm^2^ [105, 106], and relying on the hydrovoltaic effect of moisture‐enabled electrical materials. Operational life is less studied in contrast to output power and requires improvement since it is a significant limitation for practical deployment of MEGs, where most MEGs have short working times of seconds to hours for electricity generation [88, 107]. Slowing the inevitable decay of the ion concentration gradient that thermodynamically drives electricity generation is essential for prolonging the operational life of MEGs.

Photocatalysis‐assisted hydrovoltaic MEGs represent a conceptual turning point because they address this limitation at the level of non‐equilibrium physics. Conventional MEG designs implicitly assume the ion concentration gradient to be a finite, passively consumed resource. In contrast, photocatalysis‐assisted systems introduce an external energy input from light and then actively regenerate the driving gradient during operation, therefore reconstructing the operational principle of hydrovoltaic MEGs.

This strategy is exemplified by the recent research of Duan et al., who integrated a photosensitized carbon nitride (P‐CN)/PVDF‐HFP photocatalytic layer with a hygroscopic hydrogel‐based hydrovoltaic layer [19]. Crucially, the photocatalytic component does not function as a conventional photoelectric power source. Instead, it introduces a new physical role for photocatalysis in hydrovoltaic devices, i.e., light‐driven ionic pumping. This device achieved 650 h continuous operation with the photocatalytically enhanced power generation, which is 10 to 100 times the lifespan of most MEGs in literature. The photocatalysis by P‐CN under illumination is the main technical development, where electron production and chlorophyllin sodium copper salt (Chl) are processed when light sources project irradiation on the surface of P‐CN, generating electrons and holes (Equation 3). The generated electrons then convert H+ to hydrogen by the consumption of hydrogen ions (Equation 4). In the meantime, some electrons react with dissolved oxygen to generate superoxide radicals while holes react with H_2_O to generate hydroxyl radicals. The reaction scheme can be seen as below:

(3) $$CN+Chl+hv\to e^{-}+h^{+}$$

(4) $$e^{-}+2H^{+}\to H_{2}$$

(5) $$e^{-}+O_{2}\to O_{2}^{-}$$

(6) $$h^{+}+H_{2}O\to OH+H^{+}$$

Figure 7o illustrates the mechanism of utilizing a photocatalytic layer (PCL) for rebuilding ion concentration gradient and its verification. With illumination applied, PCL can increase the electrical output recovery from 0.75 to 2.2 µA and effectively influence the output performance after the illumination state [19]. Hydrovoltaic generator can operate in an open environment and act as a sustained systems through external energy coupling, overturning the prevailing assumption that MEGs must inherently function as short‐lived, disposable devices.

## 6 Applications: Contemporary Utilization and Prospective Developments

Compared with other electricity generation technologies, MEGs have still been confined to lab‐scale demonstrations with less than a decade of development [46]. Nevertheless, their exceptional potential for production to meet the ambitious target of replacing batteries in portable electronics has received increasing worldwide attention [108]. Noticing the value of these novel energy harvesting materials, recent developments cover various aspects, including the emergence of novel active membranes, promoted techniques to improve output performance, and the potential for application to broader application fields [109]. The applications of MEGs are significantly determined by their output types, e.g., transient or continuous, which are relevant to the hydroelectric mechanism and the form of moisture input, including relative humidity or changes in humidity. Continuous electrical output can be generated from the mobile ions in the EDL, whereas transient outputs of MEGs are mostly caused by the ion concentration gradient and the unstable external humidity, with experimental evidence that the output duration can vary from minutes to a monthly period [110]. The early MEGs that relied on ion concentration gradients generated from absorption‐desorption cycles or droplet evaporation could not produce a long and steady output, lasting only seconds to hours [111]. The longest reported output duration to date is 1240 h under ambient humidity (93% RH), utilizing a CNT/anodized aluminium oxide (AAO) membrane [112]. Attracted by the transience and quick response rate, MEGs have motivated contemporary adoptions and brought the potential for diverse future applications.

### 6.1 Current Applications in Self‐Powered Sensors and Power Generators

MEGs have been employed as self‐powered sensors or as energy sources for powering small electronics to eliminate the need for batteries [110]. The immediate usage of the voltage and current output of an MEG can be directly transferred to monitor humidity levels without assistance from an external power supply [89]. However, moisture‐triggered self‐powered sensors strongly rely on surroundings characterized by stable humidity levels, which has resulted in challenges when humidity conditions vary. This limitation can be remedied by employing other triboelectric or piezoelectric devices as the supplementary power source to MEGs [113]. Table 3 summarizes the output performance of MEGs as self‐powered sensors. Based on the data presented in Table 3, the maximum power density of an MEG can reach 1312 µW cm^−2^ with applications ranging from athletic suits to commercial wearable sensors. Referring to the innovations devised in the years before 2023, the most notable development in MEG‐based sensors was recorded in 2022, a MXene‐based power generator designed by Bae et al. [114]. The device could maximize electrokinetic energy conversion up to the mW scale and realized a predominant power density of 371 µW cm^−2^. The excellent output performance has been exceeded since 2023. For example, the combination of carbonized polymer dots (CPDs) and liquid metal active electrodes utilized in Li et al.’s MEG can generate high power density but was strictly confined by environmental conditions due to the metal reactivity, since common liquid metals can easily oxidize when exposed to air or moisture [115]. Feng et al. developed another attractive high‐strength sensor using poly(4‐styrenesulfonic acid) integrated with metal electrodes, demonstrating an unparalleled sensitivity [116]. The device was characterized by an ultra‐fast tactile sensing capability—a remarkable response time of 0.05 s and a superior power density of 874 µW·cm^−2^. The excellent hydrophilicity and stable ion gradient enabled the MEG to be applied in respiration and sweat monitoring, and non‐contact and ultra‐fast tactile sensing across diverse environments.

**TABLE 3:** Summary of the output performance (Open circuit voltage *U*_oc_, short circuit current *I*_sc_ and the maximum power density) of MEG devices used as self‐powered sensors under the ambient RH and temperature.

Material type	Hydrophilic material	*U*_OC_ (mV)	*I*_SC_ (µA)	Maximum power density (µW·cm^−2^)	Refs.
Carbon nanomaterial‐based	GO/rGO	760	145	15.8	[5]
Hygroscopic polymer	PSS	1050	105	874	[116]
Hydrogel	Acrylamide hydrogel	300	50	0.7	[117]
Hybrid nanomaterial‐based	GO‐ZnO	450	0.145	0.065	[118]
Fully printed	PDDA‐PSSA	1100	3.39	2.6	[119]
Carbon‐nanomaterial based	GO/PVA/PEG	600	610	384	[120]
MXene aerogel‐hydrogel	Ti3C2Tx‐PAM	600	1200	24.8	[4]
Carbon nanomaterial‐based	PA‐CPDs	800	820	1312	[115]
Carbon nanomaterial‐based	PDMS‐GO	—	—	8.64	[121]
Carbon nanomaterial‐based	PGA‐CNFs	800	22	—	[122]
Hydrogel	AMPS‐AAM	810	120	53.3	[123]
Hygroscopic polymer	PVA/NCP/MgCl2	600	2.3	0.69	[124]
Carbon nanomaterial‐based	NaCl/OH‐MWCNTs	1320	23.1	20.52	[125]
Aerogel	CMC‐single‐walled carbon nanotube	668	6.4	0.87	[126]
Hydrogel	PDDA‐PSS	800	716.25	286.5	[1]
Silicon nanowires	SiNWs	200	0.037	—	[15]
Protein	β‐lactoglobulin protein	1450	11.3	11.6	[76]
Nanocomposite	PVDF‐HFP/nano Al2O3	1030	23	10	[127]
Hygroscopic polymer	PDMM/TOCNFs/LiCl	540	—	—	[128]
Electrospun nanofibre	PAN	800	4.2	0.61	[105]

Table 4 summarizes some recent MEGs employed as power sources. The alginate‐based supramolecular hydrogel designed by Yang and colleagues can generate a comparatively high power density of over 110 µW cm^−2^ [9]. Sodium alginate is primarily derived from natural brown seaweed; the extraction process involves alkaline solution treatment followed by filtration, precipitation, and further purification. Chen et al. developed a sustainable hybrid aerogel‐hydrogel system to work for sustained power generation in environmental monitoring. The outputs reached a peak power density of 809 µWh cm^−2^ and a short circuit current of 1695 µA. Most importantly, the device can keep a stable supply for as long as 16 days without external water and over 45 days with additional moisture [129]. Compared with the applications of self‐powered sensors listed in Table 3, Table 4 emphasizes more sustainable use of materials. The remarkable MEG innovations of the past three years have shifted their trajectory to approach a more environmentally sustainable energy supply, with the potential to replace conventional fossil fuel‐based polymers. The functional materials can be composites of organic, inorganic, and biological materials. The superior characteristics that balance both high current & power densities and green and eco‐friendly derivation processes are emphasized as a critical factor in the development of next‐generation MEGs.

**TABLE 4:** Summary of the output performance of MEG devices used as power sources under the ambient RH and temperature.

Material type	Hydrophilic material	*U*_OC_ (mV)	*I*_SC_ (µA)	Maximum power density (µW·cm^−2^)	Refs.
Carbon filler‐based	PEDOT:PSS/PA‐CBW	730	360	—	[130]
Hydrogel	PVA‐AlgNa	1300	250	110	[9]
Hygroscopic polymer	PU@PSS and PEDOT:PSS	1.25	150	187.5	[2]
Hygroscopic polymer	(GO)PANI	900	126	16	[131]
Hydrogel MEG‐TENG	MXene/organo‐ionic	300	100	83	[17]
Hydrogel	PVDF‐HFP	880	306	51	[132]
Electrospun nanofibre membrane	SAlignin‐PAN	280	0.12	47	[133]
Hydrogel	PPC/Li‐PMC	1000	60	26.5	[93]
Cellulose fabric	CNW‐LiCl; PAN; P(VDF‐TrFE)	850	256	14.4	[35]
Hygroscopic polymer	Nb_2_CT_x_/HA	700	7.6	0.96	[134]
Cellulose nanofibres	CNF	250	0.11	0.2	[135]
Hygroscopic polymer	PFPE polymer brushes	1100	8.15	0.36	[136]
Carbon filler doped nonwoven	CB‐NWF	880	37.58	2.54	[137]
Hybrid system	Pd/Pd_x_O_y_ @C	300	0.162	0.17	[138]
Hygroscopic polymer	NaCl/HNT	537	17	2.44	[139]
Hybrid system	Cs_3_Bi_2_Br_9_	257	25	—	[140]
Hydrogel	PSS/GO/GI/PVA@Fab	550	7.08	1.14	[141]
Hybrid system	PAM‐SiNWs	1280	5.90	1.2	[37]
Hygroscopic polymer	H‐PSS	800	30	13	[142]
Hygroscopic polymer	PSS; nanoconfined PIL	600	45	1.6	[143]
Aero‐hydrogel	PCP@GCCP	623	1695	13.48	[129]

### 6.2 Future Perspective

**FIGURE 8:**
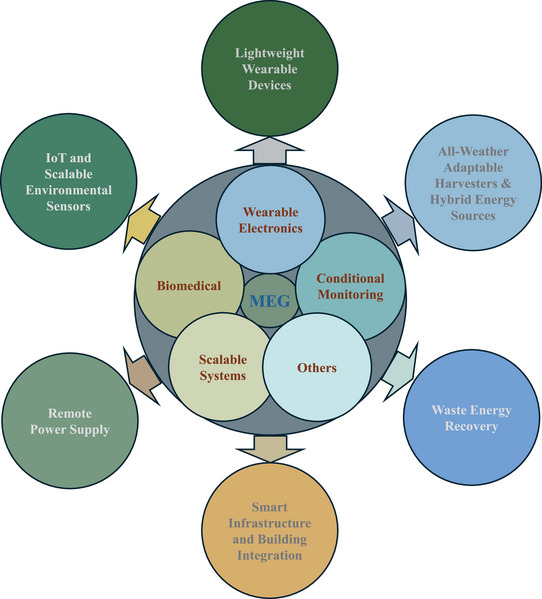
The state‐of‐the‐art and future applications of MEGs.

This review presented distinguished research on improving the energy harvesting capacity of moisture‐electric nanogenerators and the effects of material enhancement on electrical output. Though fundamental studies and lab demonstrations of MEGs have made tremendous progress, large‐scale and more environmentally adaptive operations require more work in the future. In addition, more innovation in hygroscopic functional materials and the search for extended application areas are needed for MEGs to reach their full potential. Figure 8 illustrates some current and future MEG applications that have high potential to transform the field of healthcare, environmental monitoring, and smart power systems for waste energy recycling and distributed energy supply.

**FIGURE 9:**
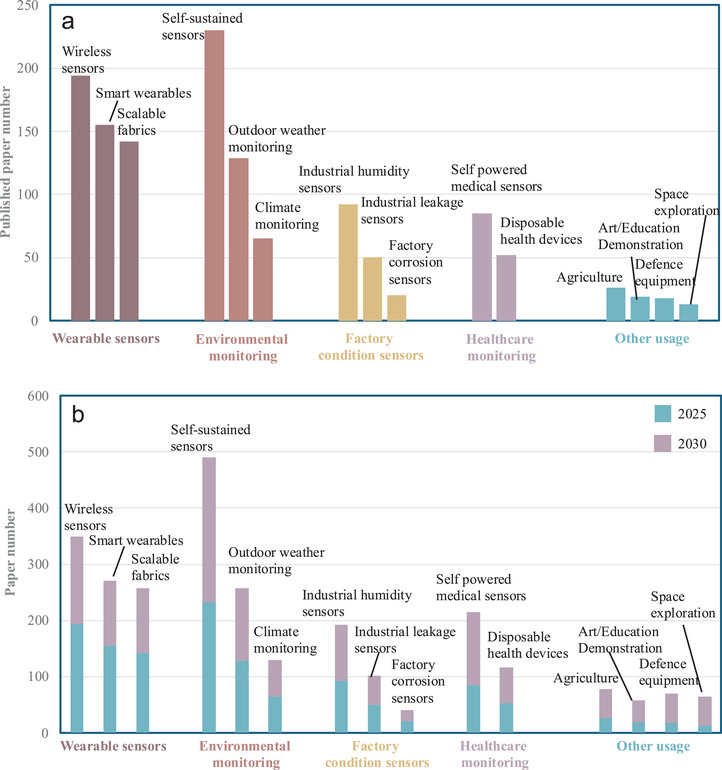
(a) Summary of papers published from 2023 to 2025 based on the categories of prevalent MEG applications. (b) The prediction of increasing publications from 2025 to 2030 using grey forecasting GM (1,1) models.

From the >2000 publications on the developments of MEG since 2023, the main application fields can be classified into wearable sensors, environmental monitoring, supplementary energy sources, healthcare monitoring, and other usage such as infrastructure‐integrated smart energy harvesting systems. The publication numbers of each field are presented in Figure 9a. Multiple grey forecasting models (GM (1,1)) [144] were constructed to predict the future trend of MEGs in the next five years. Data forecasting was generated based on the published data of MEG applications from 2023 to 2025, with each array forecasted using GM (1,1) to deduce the final volume in 2030. The inputs in each application field from 2023 to 2025 were given in raw data arrays GM^1^ (1,1) to GM^15^ (1,1) (shown in Table 5). In each GM (1,1) model, the development coefficient (*a*) and grey action quantity (*b*) are given through:

(7) $${\left(a,b\right)}^{T}={\left(B^{T}B\right)}^{-1}\;B^{T}Y$$

where matrixes B and Y are defined as:

(8) $$B=\left[\begin{array}{cc} -z^{\left(1\right)}\left(2\right) & 1\\ -z^{\left(1\right)}\left(3\right) & 1\\ \vdots & \vdots \\ -z^{\left(1\right)}\left(n\right) & 1 \end{array}\right],\;\;Y=\left[\begin{array}{c} x^{\left(0\right)}\left(2\right)\\ x^{\left(0\right)}\left(3\right)\\ \vdots \\ x^{\left(0\right)}\left(n\right) \end{array}\right]\;$$

*x* ^(0)^(*k*) is the characteristic variable (white result) of the system, which is derived from the original input data. *z* ^(1)^(*k*) is the mean generation of consecutive neighbors sequence of the accumulating generation operator sequence *x* ^(1)^(*k*) [144].

(9) $$z^{1}\;\left(k\right)=0.5\times \left(x^{\left(1\right)}\left(k\right)-x^{\left(1\right)}\left(k-1\right)\right),k=2,3,\text{…},n$$

(10) $$x^{\left(1\right)}\;\left(k\right)=\sum_{i=1}^{k}x^{\left(0\right)}\left(k\right)\;,k=1,2,\text{…},n$$

**TABLE 5:** Grey forecast model GM (1,1) inputs, original data of 2023–2025 published works.

GM∖Y	2023	2024	2025
GM1(1, 1)	48	112	197
GM2(1, 1)	37	86	151
GM3(1, 1)	35	82	144
GM4(1, 1)	55	128	227
GM5(1, 1)	31	73	131
GM6(1, 1)	15	36	65
GM7(1, 1)	22	52	94
GM8(1, 1)	12	28	50
GM9(1, 1)	5	12	22
GM10(1, 1)	18	44	80
GM11(1, 1)	12	28	51
GM12(1, 1)	6	14	26
GM13(1, 1)	4	11	20
GM14(1, 1)	4	10	19
GM15(1, 1)	3	8	15

* Column 2024 and 2025 calculated accumulation works since 2023. Column 2023 only recorded published works in the single year of 2023.

The projection of the increase in the use of MEGs has been expected and listed in the five major application fields. The maximum forecasted growth ratio in 2030 is expected to reach over 5 times in the strategic fields of military/defence systems and space exploration (Figure 9b).

*Lightweight Wearable Devices* ‒ While current developments of high‐performing MEGs have achieved significant breakthroughs in improving the power outputs, the weight reduction, environmentally harmful materials, and limited movement and portability prohibit MEGs from being applied in broader applications. In particular, MEG array systems that connect multiple MEG units in series have shown to be effective in increasing the electrical outputs [119]. However, this will increase the weight of the system and may limit applications where movement and portability are essential, e.g., wearable sensors. To conquer these barriers, increasing research efforts are required for lightweight flexible designs [145], environmentally friendly and biocompatible functional materials for monitoring human health status or training athletes. In some newly developed wearable devices, the recent advanced application incorporates Bluetooth low power (BLE) data transmission in the humidity‐driven system. Li et al. realized this through inserting a Bluetooth wireless chipset, and conducted the BLE operating current test and the BLE firmware and APP software development to make this design feasible [120].

*All‐Weather Adaptable Harvester & Hybrid Energy Sources* ‒ Climate‐specific customization has become an emerging demand, triggering improvement in the weather adaptability of MEGs [146, 147, 148]. Experimental studies conducted under various humidity conditions have demonstrated that the change of RH levels can significantly influence the outputs [18, 23, 24, 25, 26], for example, the maximum output voltage can reach almost 30 times the value of the minimum output voltage under different RH levels [18]. The humidity sensitivity drives the development of innovative weather‐adaptable MEGs. Hybrid energy systems can mutually compensate for the demerits led by a single energy conversion system through combining the MEG with another energy conversion system, such as triboelectric generation [149]. The World Health Organization recognized obstructive sleep apnea‐hypopnea syndrome (OSAHS) as one of the most prevalent organic sleep disorders [150], which inspired real‐time monitoring of respiratory status to identify sleep apnea symptoms and provide alerts, diagnosis, and appropriate treatment. It can be realised through integrating sleep apnea monitoring system in a hybrid MEG system. Yang et al. successfully developed an OSAHS system based on MEG to monitor hypopnea and apnea in real time and diagnose data for early warning [118].

*Waste Water Energy Recovery* ‒ MEG applications extend beyond simply harvesting electricity from moisture, with advanced developments in activated carbon‐based MEGs making them exceptionally compatible for generating electricity from moisture in environments contaminated with organic pollutants and heavy metals. The superior recycling capacity makes activated carbon‐based MEGs an ideal hygroscopic generator applied in wastewater treatment. While challenges remain in excluding impurities that will not facilitate the generation of ionic charges, MEGs have presented high potential in enhancing the harvesting ability when installed in wastewater facilities [151]. For instance, microbial MEGs exhibit stable outputs and durable performance under fluctuating humidity conditions, indicating that integrating hydrogels with bacterial cells in MEGs can be highly effective for realizing renewable power generation in waste anammox activated sludge [152].

*Smart Infrastructure and Building Integration* ‒ Benefiting from the moisture‐electric generation mechanisms, MEGs can be seamlessly integrated into building materials and structures, enabling buildings to autonomously harvest energy from humid air, rain or condensation. Integrating MEGs with the existing water supply system of a building can amplify the moisture‐induced potential because of the high RH level and continuous moisture interaction. This integration promotes a more self‐sustaining building power supply. In smart infrastructure applications, incorporating MEGs can not only contribute to decentralized and sustainable energy generation but also motivate real‐time sensing and adaptive control systems. These technologies can realize low‐power monitoring under indoor conditions, structural diagnostics, and autonomous humidity controls, significantly reducing dependence on external power supply and raising resilience.

*Remote power supply* ‒ The access to a reliable power source in remote, off‐grid or extreme environments has become a critical global challenge, especially for disaster response and remote connectivity. MEGs offer a possibility to operate without the demand of sunlight, fuel or large power infrastructure, utilizing water molecules absorbed from the surrounding environment instead. This feature makes them particularly suitable for the consecutive operation cycles in regions with high humidity, frequent rainfall or catenated condensation cycles. The concept of embedding MEGs into communication nodes enables them to serve as backup power for GPS units, remote control alert systems, or on‐body medical sensors under scenarios where conventional energy sources are unavailable. Furthermore, MEGs are expected to provide more powerful energy supply when combined with other energy‐harvesting technologies like thermoelectric, piezoelectric or triboelectric generation to construct hybrid nanostructures for remote deployments. Interacting electromagnetism and moisture energy harvester has been a popular research platform for powering and operating intelligent devices. Interaction between electromagnetic and moisture energy, with the regulation of ionic diodes effect, can be applied in remote self‐powered wireless charging. The all‐in‐one device, coupling with electromagnetic‐moist effect and the wireless energy interactive system, has been developed in Gao's work. It bridges the most and electromagnetic energy through diode‐like rectifying effect [28]. This innovation demonstrated the potential of using MEGs as reliable remote power suppliers with interdisciplinary science in the near future.

*IoTs and Scalable Environmental Sensors* ‒ The internet of things promotes further development of MEGs beyond the superior hygroscopic capacity and higher outputs. MEGs can be exploited for enhancing energy autonomy for IoTs, with MEGs sustaining normal operations of self‐powered units within an ultra‐low power requirement. The pivotal synergistic principles of MEGs can be utilized in optimizing intermittent energy storage and exported to ensure reliable sensor manipulation, enhancing data accuracy through preprocessing data and filtering interference from external magnetic fields for real‐time analysis. Scalable environmental sensors are superior when utilizing moisture‐electric‐based facilities for their battery‐free operation and feasible integration. These features are predicted to reduce the currently high waste and cost of energy consumption, and to lead to more research in self‐adaptive structures through integrating MEGs with walls, roads, pipes or even soil without chemical contamination.

## 7 Conclusion

This review summarizes recent advancements in moisture‐electric nanogenerators, with a particular focus on innovations in nanomaterials and structures of MEGs and their applications. The fabrication strategies cover five main methods, including optimizing ion transmission channels, constructing heterogeneous nanostructures, enhancing streaming potential through hydration reaction, hybridizing different types of nanogenerators, and developing advanced hydrogels/aerogels. Prevailingly state‐of‐the‐art mechanisms that dominate MEG performances are summarized. Optimizing materials and structures can tackle the obstacles led by low power outputs, through methodologies including scheming ionization processes—ion dissociation, ion diffusion, and proton transport, with concomitant increased streaming potential. Selecting or developing new materials paves the way to derive better MEG performance by featuring dense nanochannels and high‐performance hygroscopic components, ranging from carbon nanofillers and metallic electrodes to hydrophilic polymers and hybrid fillers. Alternative approaches focus on fabricating EDLs to enhance surface charge density and transient currents through redox reactions, utilizing the hydrovoltaic effect, creating nanoscale pores, and synergizing dissimilar energy conversion systems. Various methodologies that can improve the energy harvesting capacity have been systematically reviewed to break existing limitations, including low ion dissociation rates, reduced proton conductivity, as well as elevated internal resistance caused by low RH levels.

Current stages of MEGs applications are classified and refined through overviewing >2000 papers published in the years from 2023 to 2025. This review inserted a grey forecasting model to deduce the growth ratio of MEGs applications in 2030 with partially transparent data based on published journals. To further promote the power outputs, lightweight MEG arrays and smart infrastructure integration are employed. Remote energy supplements and all‐weather adaptive sensors stimulate more innovations in self‐adjusting moisture‐electric composites. IoTs and scalable MEG systems will prioritize the corporation of MEGs and energy autonomy. By enhancing the synergistic effects, scalable MEG‐based systems can adapt to non‐battery operations and versatile hybridization, boosting multimodal systems for higher power demands under variable humidity conditions. MEG innovations are triggering huge interests in science and technology to continuously explore sustainable moisture energy utilization.

## Conflicts of Interest

The authors declare no conflicts of interest.

## Data Availability

The data that support the findings of this study are available from the corresponding author upon reasonable request.
